# Synthesis and Evaluation of a New Type of Small Molecule Epigenetic Modulator Containing Imidazo[1,2-*b*][1,2,4]triazole Motif

**DOI:** 10.3389/fchem.2018.00642

**Published:** 2018-12-21

**Authors:** Fan Wu, Jing Zhang, Erchang Shang, Junzhi Zhang, Xiang Li, Bing Zhu, Xiaoguang Lei

**Affiliations:** ^1^Beijing National Laboratory for Molecular Sciences, Key Laboratory of Bioorganic Chemistry and Molecular Engineering of Ministry of Education, Department of Chemical Biology, Peking-Tsinghua Center for Life Sciences, College of Chemistry and Molecular Engineering, Peking University, Beijing, China; ^2^National Laboratory of Biomacromolecules, Institute of Biophysics, Chinese Academy of Sciences, Beijing, China

**Keywords:** epigenetics, DNA methylation, gene silence, structure activity relationship, p38 MAPK pathway, Imidazo[1, 2-*b*][1, 2, 4]triazole

## Abstract

Epigenetic modifications such as DNA methylation is important for many cellular processes, such as cell differentiation and cell death. The disorder of epigenetic state is closely related to human diseases, especially cancers. DNA methylation is a well-characterized epigenetic modification which is related to gene silencing and is considered as a repressive epigenetic mark. DNA methylation caused gene repression can be derepressed by chemical agents. Small molecules targeting DNA methyltransferases, histone deacetylases, and other regulatory factors can activate genes silenced by DNA methylation. However, more and more studies have shown that histone deacetylation is not the only downstream event of DNA methylation. Some additional, unknown mechanisms that promote DNA methylation-mediated gene silencing may exist. Recently, through high-throughput screening using a 308,251-member chemical library to identify potent small molecules that derepress an EGFP reporter gene silenced by DNA methylation, we identified seven hit compounds that did not directly target bulk DNA methylation or histone acetylation. Three of them (LX-3, LX-4, LX-5) were proven to selectively activate the p38 MAPK pathway in multiple cell types. In order to identify the exact cellular targets of these compounds, we turn to work on the SAR study of LX-3 by constructing a structurally diverse chemical library based on the imidazo[1,2-*b*][1,2,4]triazole core structure via diversity-oriented synthesis. Our work provides a general approach to efficiently access diverse heterocyclic molecules with interesting epigenetic modulation activities.

## Introduction

DNA methylation is a well-characterized epigenetic modification which is related to gene silence (Cedar, 1988; Bird, 2002; Bestor et al., 2015). Studies have shown that abnormal DNA methylation is associated with many diseases, including cancers, neurodegenerative diseases and heart failure. It can be observed that the overall methylation level of tumor genome is low, while the methylation level is high at the promoter regions of tumor suppressor genes (Esteller, 2008). On the other hand, many neurological diseases, including fragile X syndrome, Friedreich's ataxia and spinal muscular atrophy are associated with hypermethylation on the promoter regions of certain genes (Verkerk et al., 1991; Lefebvre et al., 1995; Campuzano et al., 1996). Unlike mutations, DNA methylations are reversible. It is possible to re-express DNA-methylated genes and to rescue their functionality by using small molecules to reactivate genes silenced by DNA methylation (Taunton et al., 1996).

**Figure 1:**
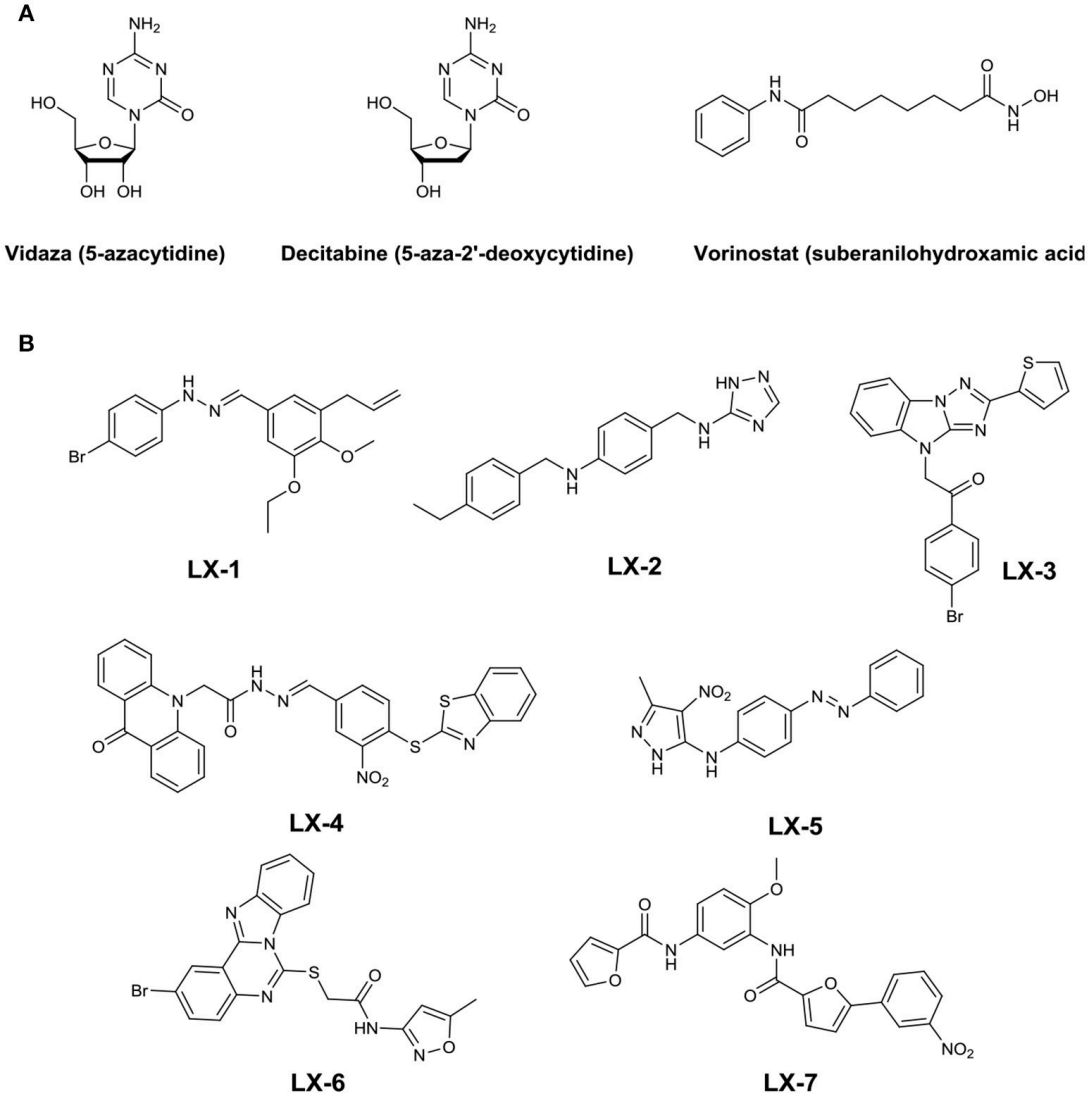
**(A)** Three anticancer drugs approved by FDA targeting epigenetic pathways. **(B)** Structures of seven hit compounds.

Early studies have shown that gene silencing was caused by DNA methyltransferases (DNMTs) and methyl-CpG(5′-Cytosine-phosphate-Guanine-3′) -binding domain proteins (MBDs) which can recruit histone deacetylases (HDACs) (Bird, 2002). Enzymes involved in this process have been identified as potential therapeutic targets. Chemical tools to modulate the functions of these proteins have been identified mainly for DNMTs and HDACs. For example, azacytidine (Vidaza) and 5-aza-2′-deoxycytidine (decitabine), inhibitors of DNMTs, havebeen approved as effective treatments for the myelodysplastic syndrome and leukemia. And the small molecule inhibitor of HDACs, Vorinostat (suberanilohydroxamic acid), has been approved by the Food and Drug Administration for the treatment of cutaneous T-cell lymphoma (Figure 1A) (Byrd et al., 2005; O'Connor et al., 2006; Garcia et al., 2010).

Accordingly, the studies on the relationship between DNA methylation and gene silencing is an important direction in the field of epigenetics. Although DNA methylation in the promoter region can inhibit the expression of the gene, it is not the major cause of gene silencing. Changes in chromatin structure resulted from a series of downstream events of DNA are the primary cause of gene silencing. DNA methylation recruits methylated CpG to recognize MBD and then HDAC, which catalyzes the deacetylation of chromatin nucleosomes in this region, makes chromatin compact. Compact chromatin rejects transcription related proteins, giving rise to gene silencing (Robertson and Wolffe, 2000).

However, this model is not complete. First, HDAC inhibitors cannot activate many DNA methylated silencing genes. In addition, HDAC inhibitor and DNA methyltransferases inhibitor showed certain complementary synergistic effect. This leads us to speculate that there may be a parallel downstream event of DNA methylation distinct from histone deacetylation. The discovery of these unknown signaling pathways may reveal a deeper understanding for DNA methylation mediated gene silencing. In order to serve the purpose of further understanding the detailed mechanism of DNA methylation-dependent gene silencing, we plan to identify novel compounds. While not directly relating to the inhibition of DNMTs and HDACs, those compounds should be able to derepress certain genes repressed by DNA methylation.

To identify novel small molecules targeting gene repression, we devised an image-based high-throughput screening system using the engineered HEK 293 F cell (termed as B2-17 cells) as report cell line. A chemical library containing 308,251 small molecules was screened in this system. Purposed for unbiased compound screening, this library had been used in a variety of studies (Sun et al., 2012; Wang et al., 2013; Dong et al., 2015; Li et al., 2016). Hundred and fifty eight compounds which reactivated reporter genes in more than half of cells were selected as hits. Next, we performed titration experiments for all these hits in the B2-17 cell line. The efficacy of each compound was evaluated by its maximal percentage of EGFP^+^ cells in the dose-response curve (maximal R_GFP_) and relative half maximal effect concentration (EC_50_). The toxicity of the hits was also tested in parallel, measured by number of viable cells in each treatment. The assay is based on quantitative analysis of ATP, an indicator of metabolically active cells. For each compound, we calculated the relative half maximal growth inhibitory concentration (IC50), based on the abovementioned assay.

**Figure 2:**
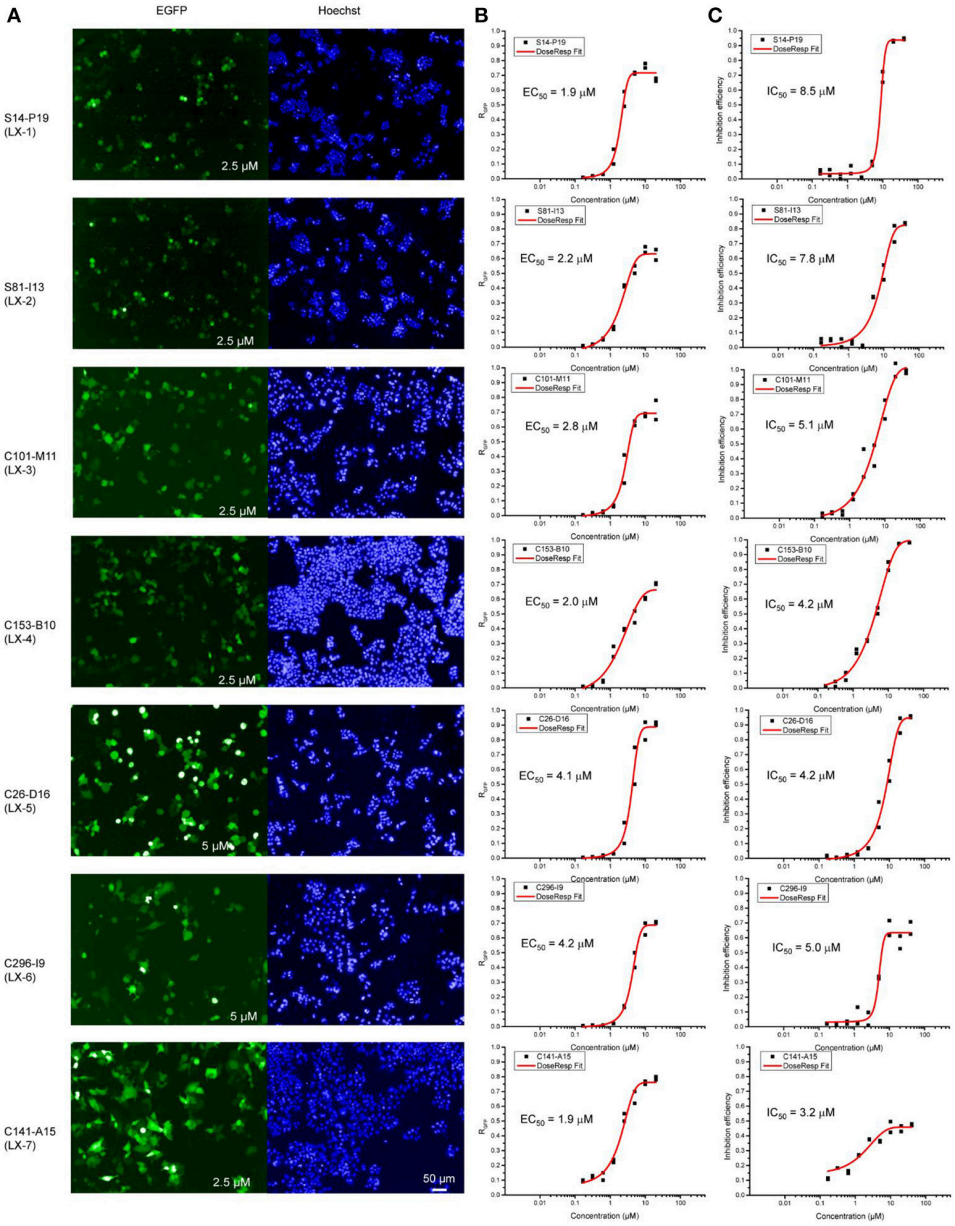
Opera confocal images **(A)**, Dose-response curve **(B)**, and lethality curve **(C)** of B2-17 cells for compound LX-1 to LX-7. The concentration used is indicated in corner of images. The EC_50_ and IC_50_ of each compound are displayed.

Seven compounds (LX-1 to LX-7) (Figure 1B) was identified following the criteria: maximal R_GFP_ >60% (good response rate), EC_50_ < 5 μM (good efficacy), and IC_50_ >3 μM (less toxic) (Figure 2), and the proof of absence of those compounds in DNA methylation or histone deacetylation levels were confirmed by subsequent biological evaluations(Dong et al., 2018; Li et al., 2018). Their relative half maximal effect concentrations (EC_50_) are 1.9, 2.2, 2.8, 2.0, 4.1, 4.2, and 1.9 μM respectively, while the relative half maximal growth inhibitory concentration (IC_50_) in turn are 8.5, 7.8, 5.1, 4.2, 4.2, 5.0, and 3.2 μM respectively (Table 1). The majority of genes upregulated by compound LX-3, LX-4, and LX-5 overlapped with each other, suggesting that these compounds may function through the same pathway. Results from Gene Ontology (GO) enrichment analysis suggested that the DNA derepression effect of compound LX-3, LX-4, and LX-5 are likely to functionalize via the activation of the MAPKs pathway and its downstream transcription factors. Further study shows these compounds selectively activate the p38 MAPK pathway, which is capable of activating the methylated EGFP reporter gene.

**Table 1:** Bioactivity data for seven lead compounds.

**No**.	**EC_**50**_ (μM)**	**Maximal R_**GFP**_%**	**IC_**50**_ (μM)**
LX-1	1.9	72	8.5
LX-2	2.2	67	7.8
LX-3	2.8	79	5.1
LX-4	2.0	68	4.2
LX-5	4.1	89	4.2
LX-6	4.2	71	5.0
LX-7	1.9	79	3.2

In general, these results show that LX-3, LX-4, and LX-5 are related with p38 MAPK pathway as agonists, but the exact targets of these compounds are elusive. In order to generate more potent and selective compounds to develop effective chemical probes for target identification, LX-3 that possessed novel structure and potent activity was herein chosen as our lead compound for the following structure-activity relationship (SAR) studies.

**Scheme 1:**
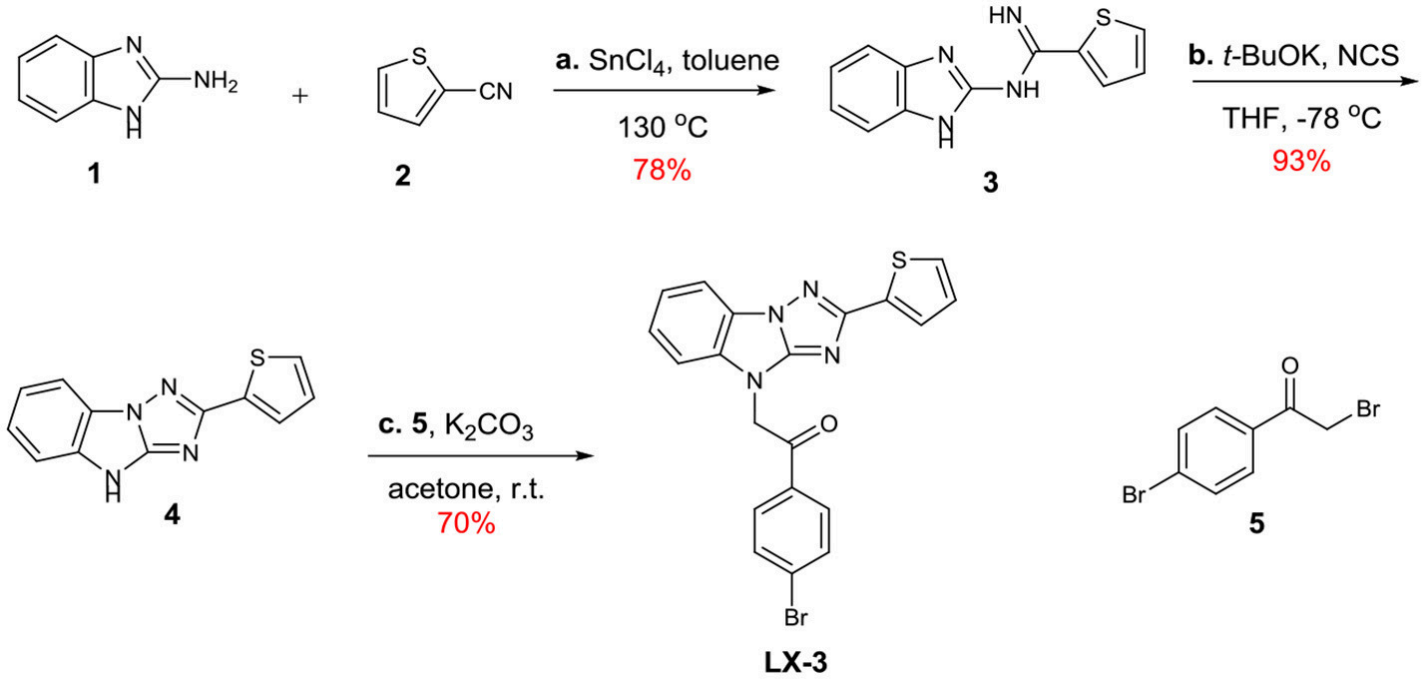
Synthesis of the hit compound **LX-3**. General conditions: (a) SnCl_4_, toluene, 130 °C (b) *t*-BuOK, NCS, THF, −78°C (c) K_2_CO_3_, acetone, r.t. *t*-BuOK = Potassium tert-butoxide NCS = N-chlorosuccinimide.

Diversity-oriented synthesis (DOS) has proven to be a successful strategy for the rapid access to structurally diverse chemical libraries (Galloway et al., 2010). A DOS library was designed based on the central imidazo[1,2-*b*][1,2,4]triazole (Figure 2). Around it, compound LX-3 can be divided into three parts (labeled as A–C respectively). A is a benzene ring, which is fused with the imidazole of the central structure. The right side of this molecule is connected with the thiophene ring (B) at the 2-position, and the bottom part is connected with 4′-bromoacetophenone (C) at the 4-position. The aromatic ring of C and the imidazo[1,2-*b*][1,2,4]triazole are connected by a two-carbon unit (Linker). There was no efficient synthetic strategy for such novel heterocyclic structure. Recently we developed an efficient synthetic strategy for [1,2,4]triazolo[1,5-*a*]benzazole type scaffold (Shang et al., 2016). As shown in Scheme 1, the synthesis of the key intermediate **4** is taken as an example. Commercially available materials **1** and **2** were assembled to afford guanidine compound **3** (78% yield) under the catalysis of tin tetrachloride. And under the condition of NCS and potassium *tert*-butanol, the [1,2,4]triazolo[1,5-*a*]benzazole type scaffold was obtained in high yield. Here, for the synthesis of the lead compound **LX-3**, **4** was further reacted with 2,4′-dibromoacetophenone **5** using potassium carbonate to provide the desired product smoothly. This convergent strategy was applied to install diverse A, B and C units to afford a DOS library for SAR study of **LX-3**.

**Figure 3:**
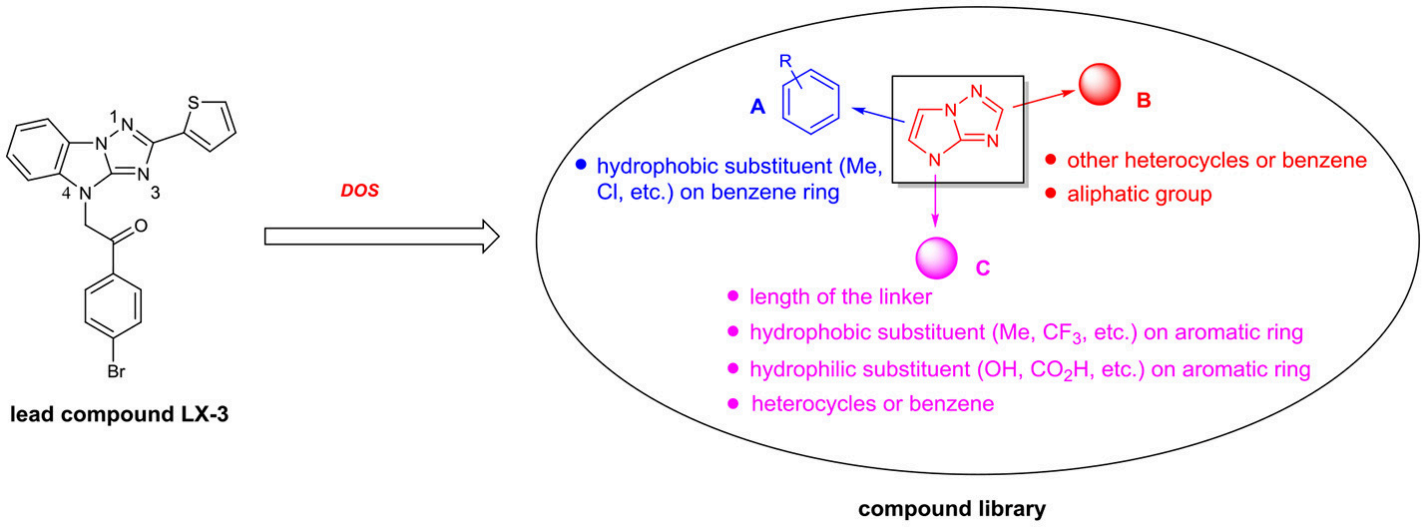
Structural analysis and design for the SAR study of the hit compound LX-3.

In terms of molecular hydrophobicity of **LX-3**, the [1,2,4]triazolo[1,5-*a*]benzazole is mainly hydrophobic (Figure 3), while nitrogen atoms at 1- and 3-positions are potential hydrogen bond acceptors. The B and C are also hydrophobic, so the overall hydrophobicity of this molecule is very high. The carbonyl group can act as a potential hydrogen bond acceptor. For the structural diversification of C, we can replace the bromine atom by a variety of hydrophobic or hydrophilic groups, such as other halogens, hydroxyl groups, carboxyl groups, etc. These functional groups can also be launched at different positions of the benzene ring. In addition, the benzene ring can be replaced by other heterocycles to evaluate the effect on the bioactivity. We can also modify the length of the linker of C. For the structural diversification of B, substituents can be introduced at different positions of the thiophene, such as methyl and chlorine atoms. The thiophene can also be replaced by other heterocycles, phenyl group or aliphatic group. The SAR of A can be investigated by introducing different hydrophobic substituents to the benzene.

## Materials and Methods

### General Information

^1^H NMR spectra were recorded on Brucker ARX 400 MHz or DRX 500 MHz spectrometer at ambient temperature with CDCl_3_ or DMSO-d6 or CD_3_OD as the solvent unless otherwise stated. Chemical shifts are reported in parts per million relative to chloroform or DMSO (1H, δ 7.26 for CDCl_3_, 2.50 for DMSO-d6, 3.31 for CD_3_OD). Data for ^1^H NMR are reported as follows: chemical shift, integration, multiplicity (s = singlet, d = doublet, t = triplet, q = quartet, m = multiplet) and coupling constants. High-resolution mass spectra were obtained at Peking University Mass Spectrometry Laboratory using a Bruker APEX Flash chromatography. Flash chromatography was performed using 200–300 mesh silica gel. Yields refer to chromatographically and spectroscopically pure materials unless otherwise stated. Dichloromethane, dichloroethane, acetonitrile, and dimethyl formamide were distilled from calcium hydride; tetrahydrofuran was distilled from sodium/benzophenone ketyl prior to use. Reagents were purchased at the highest commercial quality and used without further purification unless otherwise stated. All reactions were carried out in oven-dried glassware under an argon atmosphere with dry solvents unless otherwise noted. The compound library consisted of 5 sub-libraries purchased from Maybridge, Enamine, Specs, Chemdiv, and WXTD.

### Synthesis

**N-(1H-benzo[d]imidazol-2-yl)thiophene-2-carboximidamide 3**

General procedure A: A mixture of 1 (133.2 mg, 1 mmol), 2 (109.1 mg, 1 mmol) and anhydrous tin tetrachloride (1 M in dichloromethane, 3 mL, 3 mmol) in a sealed tube was heated at 130°C under argon for 24 h. The mixture was then stirred at room temperature and dispersed into the ethyl acetate. The obtained ethyl acetate solution was poured into the ice cold aq. 20% NaOH gradually, and the resulting mixture was extracted with ethyl acetate (20 mL × 3). The organic layers were combined together, washed with water, brine, and dried over Na_2_SO_4_ successively. After evaporation of the solvent under reduced pressure, the residue was subjected to column chromatography on silica gel column chromatography (PE/EtOAc = 1/1) to provide the 3 as a white powder(189.0 mg, 78%).

^**1**^**H NMR** (400 MHz, DMSO-d6) δ 11.93 (br, 1H), 10.27 (br, 1H), 8.68 (br, 1H), 7.93 (dd, *J* = 4.0 Hz, 1.2 Hz, 1H), 7.76 (dd, *J* = 4.0 Hz, 1.2 Hz, 1H), 7.45-7.48 (m, 1H), 7.23-7.27 (m, 1H), 7.19-7.22 (m, 1H), 7.09~7.04 (m, 2H).

**HRMS** (ESI) [M + H]^+^ calculated for C_12_H_11_N_4_S: 243.0695, found: 243.0670.

**2-(thiophen-2-yl)-4H-benzo[4,5]imidazo[1,2-b][1,2,4] triazole 4**

General procedure B: To a flame-dried 10 mL flask with a magnet, was added the amidine 3 (24.2 mg, 0.1 mmol) and KO*t*Bu (44.8 mg, 0.4 mmol) under argon. The flask was cooled in a dry ice-acetone bath, and THF (2 mL) was then added with stirring. After 1 h stirring at same temperature, a solution of N-chlorosuccinimide (NCS) (16.0 mg, 0.12 mmol) in THF (2 mL) was added and the result mixture was stirred until it finished by TLC monitor (about 0.5~1 h). The reaction was then quenched with saturated aqueous NH_4_Cl (1 mL) and moved to room temperature. The system was diluted with ethyl acetate, washed with water, brine and dried over Na_2_SO_4_ successively. The dried solution was concentrated in vacuo to 2 mL, filtrated and washed with a small amount of ethyl acetate to obtain a large portion of the final product. The mother liquid was then purified by a short silica gel column chromatography (PE/EtOAc = 1/2) to provide another portion of the final product. The two portions were combined together to afford 4 as a white powder (22.3 mg, 93%).

^**1**^**H NMR** (400 MHz, DMSO-d6) δ 12.45 (br, 1H), 7.84 (d, *J* = 8 Hz, 1H), 7.70 (dd, *J* = 4 Hz, 0.8 Hz, 1H), 7.65 (dd, *J* = 4 Hz, 0.8 Hz, 1H), 7.56 (d, *J* = 8 Hz, 1H), 7.34-7.40 (m, 1H), 7.30-7.32 (m, 1H), 7.19 (dd, *J* = 8 Hz, 4 Hz, 1H).

**HRMS** (ESI) [M + H]^+^ calculated for C_12_H_9_N_4_S: 241.0540, found: 241.0542.

**1-(4-bromophenyl)-2-(2-(thiophen-2-yl)-4H-benzo[4,5] imidazo[1,2-b][1,2,4]triazol-4-yl)ethan-1-one LX-3**

General procedure C: To a flame-dried 10 mL flask with a magnet, potassium carbonate (55.3 mg, 0.4 mmol) was added to the anhydrous acetone solution (5 mL) of 4 (24.0 mg, 0.1 mmol) and bromide 5 (30.6 mg, 0.11 mmol). The reaction mixture was stirred overnight, diluted with water and extracted with ethyl acetate. Washed with brine and then dried over Na_2_SO_4_. filtered and concentrated in vacuo to give a crude oil, which was purified by silica gel column chromatography (PE/EtOAc = 1/2) to afford compound LX-3 as a white powder (30.6 mg, 70%).

^**1**^**H NMR** (400 MHz, CDCl_3_) δ 7.93 (d, *J* = 8.4 Hz, 2H), 7.90–7.85 (m, 1H), 7.77 (d, *J* = 3.5 Hz, 1H), 7.70 (d, *J* = 8.4 Hz, 2H), 7.38 (d, *J* = 5.0 Hz, 1H), 7.37–7.30 (m, 2H), 7.22–7.17 (m, 1H), 7.15–7.11 (m, 1H), 5.66 (s, 2H);

^**13**^**C NMR** (151 MHz, CDCl_3_) δ 190.0, 161.3, 134.9, 134.5, 132.8, 132.5, 129.9, 129.6, 127.8, 126.8, 126.6, 124.7, 124.0, 122.4, 111.3, 110.6, 49.6.

**HRMS** (ESI) [M + H]^+^ calculated for C_20_H_14_BrN_4_OS: 437.0066, found: 437.0081.

**1-phenyl-2-(2-(thiophen-2-yl)-4H-benzo[4,5] imidazo[1,2-b][1,2,4]triazol-4-yl)ethan-1-one 7a**

According to the general procedure C, the reaction was finished in 12 h.

White powder,1.0 mg, yield 67% ^**1**^**H NMR** (400 MHz, CDCl_3_) δ 8.08 (d, *J* = 8.4 Hz, 2H), 7.88 (d, *J* = 4.8 Hz, 1H), 7.77 (dd, *J* = 4.0, 1.6 Hz, 1H), 7.67 (t, *J* = 5.6 Hz, 1H), 7.57 (t, *J* = 8.4 Hz, 2H), 7.38–7.33 (m, 3H), 7.23–7.20 (m, 1H), 7.13–7.11 (m, 1H), 5.72 (s, 2H).

**1-(4-methoxyphenyl)-2-(2-(thiophen-2-yl)-4H-benzo[4,5] imidazo[1,2-b][1,2,4]triazol-4-yl)ethan-1-one 7b**

According to the general procedure C, the reaction was finished in 12 h.

White powder,1.1 mg, yield 58% ^**1**^**H NMR** (400 MHz, CDCl_3_) δ 8.04 (d, *J* = 8.9 Hz, 2H), 7.89–7.84 (m, 1H), 7.77 (dd, *J* = 3.6, 1.0 Hz, 1H), 7.37 (dd, *J* = 5.0, 1.0 Hz, 1H), 7.34–7.29 (m, 2H), 7.23–7.18 (m, 1H), 7.12 (dd, *J* = 4.9, 3.7 Hz, 1H), 7.01 (d, *J* = 8.9 Hz, 2H), 5.66 (s, 2H), 3.91 (s, 3H);

^**13**^**C NMR** (151 MHz, CDCl3) δ 189.1, 164.5, 161.2, 154.5, 135.2, 134.6, 130.6, 127.7, 127.2, 126.7, 126.6, 124.6, 124.0, 122.2, 114.3, 111.2, 110.8, 55.6, 49.4.

**HRMS** (ESI) [M + H]^+^ calculated for C_21_H_17_N_4_O_2_S: 389.1067, found: 389.1075.

**1-(4-nitrophenyl)-2-(2-(thiophen-2-yl)-4H-benzo[4,5] imidazo[1,2-b][1,2,4]triazol-4-yl)ethan-1-one 7c**

According to the general procedure C, the reaction was finished in 12 h.

Yellow powder,1.4 mg, yield 20% ^**1**^**H NMR** (400 MHz, CDCl_3_) δ 8.40 (d, *J* = 8.6 Hz, 2H), 8.25 (d, *J* = 8.7 Hz, 2H), 7.92–7.83 (m, 1H), 7.77 (d, *J* = 3.5 Hz, 1H), 7.43–7.32 (m, 3H), 7.23–7.18 (m, 1H), 7.16–7.09 (m, 1H), 5.73 (s, 2H).

^**13**^**C NMR** (151 MHz, CDCl_3_) δ 189.8, 161.3, 154.3, 151.1, 138.4, 134.8, 134.4, 129.4, 127.8, 126.9, 126.7, 124.7, 124.3, 124.2, 122.6, 111.4, 110.5, 50.1.

**HRMS** (ESI) [M + H]^+^ calculated for C_20_H_14_N_5_O_3_S: 404.0812, found: 404.0802.

**methyl 4-(2-(2-(thiophen-2-yl)-4H-benzo[4,5] imidazo[1,2-b][1,2,4]triazol-4-yl)acetyl)benzoate 7d**

According to the general procedure C, the reaction was finished in 24 h.

White powder,7.1 mg, yield 30% ^**1**^**H NMR** (400 MHz, CDCl_3_) δ 8.21 (d, *J* = 8.4 Hz, 2H), 8.13 (d, *J* = 8.3 Hz, 2H), 7.91 – 7.86 (m, 1H), 7.77 (d, *J* = 3.6 Hz, 1H), 7.40–7.33 (m, 3H), 7.23–7.19 (m, 1H), 7.15–7.11 (m, 1H), 5.73 (s, 2H), 3.98 (s, 3H).

^**13**^**C NMR** (151 MHz, CDCl_3_) δ 189.9, 164,3, 159.0, 143.6, 142.7, 138.9, 134.3, 128.8, 128.5, 128.1, 127.4, 125.1, 124.1, 123.9, 123.2, 122.6, 111.9, 110.0, 51.6, 50.3.

**HRMS** (ESI) [M + H]^+^ calculated for C_22_H_17_N_4_O_3_S: 417.1016, found: 417.1015;

**2-(2-(thiophen-2-yl)-4H-benzo[4,5] imidazo[1,2-b][1,2,4]triazol-4-yl)-1-(4-(trifluoromethyl)phenyl)ethan-1-one 7e**

According to the general procedure C, the reaction was finished in 12 h.

White powder,1.1 mg, yield 17% ^**1**^**H NMR** (400 MHz, CDCl_3_) δ 8.19 (d, *J* = 8.2 Hz, 2H), 7.88 (dd, *J* = 5.3, 3.9 Hz, 1H), 7.83 (d, *J* = 8.3 Hz, 2H), 7.77 (dd, *J* = 3.6, 1.1 Hz, 1H), 7.39 (dd, *J* = 5.0, 1.1 Hz, 1H), 7.34 (dd, *J* = 6.3, 2.7 Hz, 2H), 7.22–7.18 (m, 1H), 7.13 (dd, *J* = 5.0, 3.7 Hz, 1H), 5.72 (s, 2H);

^**13**^**C NMR** (151 MHz, CDCl3) δ 190.2, 161.3, 154.4, 136.7, 134.9, 134.5, 128.6, 127.8, 126.8, 126.6, 126.2, 126.2, 124.7, 124.1, 122.5, 111.3, 110.5, 49.9.

**HRMS** (ESI) [M + H]^+^ calculated for C_21_H_14_F_3_N_4_OS: 427.0835, found: 427.0837.

**1-(2,4-difluorophenyl)-2-(2-(thiophen-2-yl)-4H-benzo[4,5]imidazo[1,2-b][1,2,4]triazol-4-yl)ethan-1-one 7f**

According to the general procedure C, the reaction was finished in 12 h at 40°C.

White powder,5.0 mg, yield 44% ^**1**^**H NMR** (400 MHz, CDCl_3_) δ 8.06 (dd, *J* = 15.0, 8.3 Hz, 1H), 7.91–7.86 (m, 1H), 7.76 (d, *J* = 3.5 Hz, 1H), 7.35 (dt, *J* = 14.9, 5.4 Hz, 3H), 7.19 (dd, *J* = 5.3, 3.8 Hz, 1H), 7.14–7.10 (m, 1H), 7.09–6.99 (m, 2H), 5.61 (d, *J* = 3.9 Hz, 2H).

^**13**^**C NMR** (151 MHz, CDCl3) δ 187.7, 142.7, 142.2, 135.0, 134.6, 133.3, 133.2, 127.7, 126.7, 126.6, 124.6, 123.9, 122.3, 113.2, 113.1, 111.3, 110.4, 105.0, 53.1.

**HRMS** (ESI) [M + H]^+^ calculated for C_20_H_13_F_2_N_4_OS: 395.0773, found: 395.0768;

**1-(perfluorophenyl)-2-(2-(thiophen-2-yl)-4H-benzo[4,5] imidazo[1,2-b][1,2,4]triazol-4-yl)ethan-1-one 7g**

According to the general procedure C, the reaction was finished in 12 h at 40°C.

White powder,2.0 mg, yield 22% ^**1**^**H NMR** (400 MHz, CDCl_3_) δ 7.88 (dd, *J* = 6.1, 3.1 Hz, 1H), 7.77 (dd, *J* = 3.6, 1.0 Hz, 1H), 7.41–7.34 (m, 3H), 7.21 (dd, *J* = 6.2, 3.0 Hz, 1H), 7.13 (dd, *J* = 5.0, 3.7 Hz, 1H), 5.52 (s, 2H).

**HRMS** (ESI) [M + H]^+^ calculated for C_20_H_10_F_5_N_4_OS: 449.0490, found: 449.0491;

**1-(thiazol-2-yl)-2-(2-(thiophen-2-yl)-4H-benzo[4,5] imidazo[1,2-b][1,2,4]triazol-4-yl)ethan-1-one 9a**

According to the general procedure C, the reaction was finished in 12 h.

White powder,3.8 mg, yield 57% ^**1**^**H NMR** (400 MHz, CDCl_3_) δ 8.15 (d, *J* = 3.0 Hz, 1H), 7.93–7.87 (m, 1H), 7.84 (d, *J* = 3.0 Hz, 1H), 7.77 (d, *J* = 3.6 Hz, 1H), 7.41–7.30 (m, 3H), 7.24 (d, *J* = 4.3 Hz, 1H), 7.16–7.09 (m, 1H), 5.92 (s, 2H);

^**13**^**C NMR** (151 MHz, CDCl_3_) δ 184.9, 163.6, 161.4, 145.4, 134.6, 135.0, 127.7, 127.5, 126.7, 126.6, 124.7, 124.0, 122.3, 111.3, 110.4, 49.6.

**HRMS** (ESI) [M + H]^+^ calculated for C_17_H_12_N_5_OS_2_: 366.0478, found: 366.0470.

**1-(furan-2-yl)-2-(2-(thiophen-2-yl)-4H-benzo[4,5] imidazo[1,2-b][1,2,4]triazol-4-yl)ethan-1-one 9b**

According to the general procedure C, the reaction was finished in 12 h.

White powder,1.9 mg, yield 14% ^**1**^**H NMR** (400 MHz, CDCl_3_) δ 7.87 (dd, *J* = 5.9, 3.2 Hz, 1H), 7.81–7.76 (m, 1H), 7.70 (d, *J* = 1.0 Hz, 1H), 7.42 (d, *J* = 3.6 Hz, 1H), 7.39–7.37 (m, 1H), 7.33 (dt, *J* = 7.4, 3.7 Hz, 2H), 7.12 (dd, *J* = 4.9, 3.7 Hz, 1H), 6.65 (dd, *J* = 3.6, 1.7 Hz, 1H), 5.57 (s, 2H);

^**13**^**C NMR** (151 MHz, CDCl3) δ 185.0, 147.4, 127.7, 126.7, 126.6, 124.6, 124.0, 122.8, 122.3, 121.8, 118.7, 113.0, 111.9, 111.2, 110.7, 109.5, 53.4.

**HRMS (ESI)** [M + H]^+^ calculated for C_18_H_13_N_4_O_2_S: 349.0754, found: 349.0751.

**1-(thiophen-2-yl)-2-(2-(thiophen-2-yl)-4H-benzo[4,5] imidazo[1,2-b][1,2,4]triazol-4-yl)ethan-1-one 9c**

According to the general procedure C, the reaction was finished in 12 h.

White powder,2.0 mg, yield 17% ^**1**^**H NMR** (400 MHz, CDCl_3_) δ 7.99 (d, *J* = 3.6 Hz, 1H), 7.87 (dd, *J* = 5.9, 3.0 Hz, 1H), 7.78 (d, *J* = 4.3 Hz, 2H), 7.41 – 7.31 (m, 3H), 7.29 (d, *J* = 3.3 Hz, 1H), 7.23 (d, *J* = 4.3 Hz, 1H), 7.16–7.09 (m, 1H), 5.61 (s, 2H);

^**13**^**C NMR** (151 MHz, CDCl_3_) δ 189.9, 158.5, 147.9, 142.5, 141.6, 134.8, 134.0, 125.2, 124.3, 124.0, 122.9, 122.1, 120.6, 118.5, 110.8, 110.1, 49.5.

**HRMS** (ESI) [M + H]^+^ calculated for C_18_H_13_N_4_OS2: 365.0525, found: 365.0532.

**1-(pyridin-4-yl)-2-(2-(thiophen-2-yl)-4H-benzo[4,5] imidazo[1,2-b][1,2,4]triazol-4-yl)ethan-1-one 9d**

According to the general procedure C, the reaction was finished in 12 h.

Yellow powder, 6.6 mg, yield 88% ^**1**^**H NMR** (400 MHz, CDCl_3_) δ 8.91 (d, *J* = 5.8 Hz, 2H), 7.91–7.82 (m, 3H), 7.79–7.75 (m, 1H), 7.41–7.31 (m, 3H), 7.21–7.16 (m, 1H), 7.13 (dd, *J* = 4.9, 3.7 Hz, 1H), 5.69 (s, 2H);

^**13**^**C NMR** (151 MHz, CDCl_3_) δ 190.9, 161.3, 151.4, 139.8, 134.8, 134.4, 127.8, 126.9, 126.7, 124.7, 124.1, 122.6, 120.9, 111.4, 110.4, 49.9.

**HRMS** (ESI) [M + H]^+^ calculated for C_19_H_13_N_5_OS: 360.0914, found: 360.0904.

**1-(4-bromophenyl)-2-(6,7-dimethyl-2-(thiophen-2-yl)-4H-benzo[4,5]imidazo[1,2-b][1,2,4]triazol-4-yl)ethan-1-one 14a**

According to the general procedure C, the reaction was finished in 12 h.

White powder,11.1 mg, yield 92% ^**1**^**H NMR** (400 MHz, CDCl_3_) δ 7.92 (d, *J* = 8.5 Hz, 2H), 7.74 (dd, *J* = 3.6, 0.9 Hz, 1H), 7.69 (d, *J* = 8.5 Hz, 2H), 7.64 (s, 1H), 7.36 (dd, *J* = 5.0, 0.9 Hz, 1H), 7.11 (dd, *J* = 4.9, 3.7 Hz, 1H), 6.94 (s, 1H), 5.60 (s, 2H), 2.38 (s, 3H), 2.34 (s, 3H);

^**13**^**C NMR** (151 MHz, CDCl_3_) δ 189.5, 153.7, 146.3, 142.5, 134.8, 133.5, 132.0, 131.1, 129.8, 129.0, 128.2, 127.1, 123.8, 119.1, 116.4, 111,0, 49.5, 18.1, 17.5.

**HRMS** (ESI) [M + H]^+^ calculated for C_22_H_18_BrN_4_OS: 465.0379, found: 465.0377.

**1-(4-bromophenyl)-2-(6,7-dimethyl-2-(thiazol-2-yl)-4H-benzo[4,5] imidazo[1,2-b][1,2,4]triazol-4-yl)ethan-1-one 14b**

According to the general procedure C, the reaction was finished in 12 h.

White powder,2.7 mg, yield 29% ^**1**^**H NMR** (400 MHz, CDCl_3_) δ 7.94 (dd, *J* = 12.9, 5.8 Hz, 3H), 7.70 (d, *J* = 8.4 Hz, 3H), 7.42 (d, *J* = 3.0 Hz, 1H), 6.99 (s, 1H), 5.65 (s, 2H), 2.41 (s, 3H), 2.36 (s, 3H).

^**13**^**C NMR** (151 MHz, CDCl_3_) δ 190.1, 154.3, 144.0, 141.1, 133.6, 132.7, 131.5, 131.0, 129.7, 127.3, 125.9, 123.0, 118.7, 116.4, 111,3, 50.1, 17.9, 16.7.

**HRMS** (ESI) [M + H]^+^ calculated for C_21_H_17_BrN_5_OS: 466.0332, found: 466.0326.

**2-(7-bromo-2-(thiophen-2-yl)-4H-benzo[4,5] imidazo[1,2-b][1,2,4]triazol-4-yl)-1-(4-bromophenyl)ethan-1-one 17a**

According to the general procedure C, the reaction was finished in 6 h.

White powder,4.2 mg, yield 24% ^**1**^**H NMR** (400 MHz, CDCl_3_) δ 8.03 (s, 1H), 7.93 (d, *J* = 8.5 Hz, 2H), 7.76 (d, *J* = 3.6 Hz, 1H), 7.71 (d, *J* = 8.5 Hz, 2H), 7.47 – 7.43 (m, 1H), 7.39 (d, *J* = 5.0 Hz, 1H), 7.15 – 7.10 (m, 1H), 7.07 (d, *J* = 8.7 Hz, 1H), 5.65 (s, 2H);

**HRMS** (ESI) [M + H]^+^ calculated for C_20_H_13_Br_2_N_4_OS: 514.9171, found: 514.9159.

**2-(7-bromo-2-(5-bromothiophen-2-yl)-4H-benzo[4,5] imidazo[1,2-b][1,2,4]triazol-4-yl)-1-(4-bromophenyl)ethan-1-one 17c**

According to the general procedure C, the reaction was finished in 4 h.

White powder,3.0 mg, yield 15% ^**1**^**H NMR** (500 MHz, CDCl_3_) δ 8.02 (d, *J* = 1.6 Hz, 1H), 7.92 (d, *J* = 8.5 Hz, 2H), 7.71 (d, *J* = 8.5 Hz, 2H), 7.49 (d, *J* = 3.9 Hz, 1H), 7.45 (dd, *J* = 8.6, 1.7 Hz, 1H), 7.08 (d, *J* = 4.1 Hz, 1H), 7.06 (s, 1H), 5.62 (s, 2H).

^**13**^**C NMR** (151 MHz, CDCl_3_) δ 189.5, 135.7, 132.7, 132.6, 130.7, 130.1, 129.6, 127.3, 127.1, 126.9, 125.5, 125.3, 115.2, 114.5, 114.3, 113.0, 111.9, 49.7.

**HRMS** (ESI) [M + H]^+^ calculated for C_20_H_12_Br_3_N_4_OS: 592.8276, found: 592.8277.

**1-(4-bromophenyl)-2-(7-chloro-2-(thiophen-2-yl)-4H-benzo[4,5]imidazo[1,2-b][1,2,4]triazol-4-yl)ethan-1-one 19**

To a solution of 16a (3.0 mg, 0.0094 mmol) in DMF was added CuCl (1.9 mg, 0.0188 mmol), the reaction was stirred at 160°C for 6 h, then cooled to r.t. Ethyl acetate was added and the resulting solution was stirred with saturated aq. NH_4_Cl/ NH_3_·H_2_O (9:1) solution for 15 min. Then extracted with ethyl acetate. The organic layer was dried over Na_2_SO_4_, filtered and concentrated in vacuo to yield the crude product and used directly in next step.

According to the general procedure C, the reaction was finished in 5 h.

White powder,1.5 mg, 34% ^**1**^**H NMR** (400 MHz, CDCl_3_) δ 7.93 (d, *J* = 8.6 Hz, 2H), 7.88 (d, *J* = 1.9 Hz, 1H), 7.76 (d, *J* = 2.6 Hz, 1H), 7.71 (d, *J* = 8.6 Hz, 2H), 7.40 (d, *J* = 5.0 Hz, 1H), 7.31 (dd, *J* = 8.7, 1.9 Hz, 1H), 7.12 (dd, *J* = 8.6, 4.5 Hz, 2H), 5.65 (s, 2H);

**HRMS** (ESI) [M + H]^+^ calculated for C_20_H_13_BrClN_4_OS: 470.9676, found: 470.9666.

**1-(4-bromophenyl)-2-(2-phenyl-4H-benzo[4,5] imidazo[1,2-b][1,2,4]triazol-4-yl)ethan-1-one 22a**

According to the general procedure C, the reaction was finished in 12 h.

White powder,41.1 mg, yield 54% ^**1**^**H NMR** (400 MHz, CDCl_3_) δ 8.22–8.16 (m, 2H), 7.96 (d, *J* = 8.5 Hz, 2H), 7.92–7.86 (m, 1H), 7.71 (d, *J* = 8.5 Hz, 2H), 7.45 (ddd, *J* = 8.5, 7.7, 2.2 Hz, 3H), 7.38–7.30 (m, 2H), 7.24–7.19 (m, 1H), 5.68 (s, 2H).

^**13**^**C NMR** (151 MHz, CDCl3) δ 190.2, 165.5, 154.7, 135.0, 132.9, 132.5, 131.7, 129.7, 129.5, 128.6, 126.7, 124.0, 122.3, 111.3, 110.6, 100.0, 49.7.

**HRMS** (ESI) [M + H]^+^ calculated for C_22_H_16_BrN_4_O: 431.0502, found: 431.0492.

**1-(4-bromophenyl)-2-(2-(2-methoxyphenyl)-4H-benzo[4,5] imidazo[1,2-b][1,2,4]triazol-4-yl)ethan-1-one 22b**

According to the general procedure C, the reaction was finished in 12 h.

White powder,10.4 mg, yield 87% ^**1**^**H NMR** (400 MHz, CDCl_3_) δ 8.00–7.90 (m, 4H), 7.69 (d, *J* = 8.6 Hz, 2H), 7.42 (dd, *J* = 11.7, 4.0 Hz, 1H), 7.34 (dd, *J* = 6.4, 2.8 Hz, 2H), 7.23–7.19 (m, 1H), 7.06 (dd, *J* = 12.1, 4.7 Hz, 2H), 5.68 (s, 2H), 3.98 (s, 3H);

^**13**^**C NMR** (151 MHz, CDCl_3_) δ 190.0, 159.4, 158.0, 141.3, 133.6, 131.4, 131.0, 129.5, 128.9, 128.2, 127.8, 124.0, 123.8, 122.9, 121.3, 120.5, 110.4, 109.6, 53.4, 49.3.

**HRMS** (ESI) [M + H]^+^ calculated for C_23_H_18_BrN_4_O_2_: 461.0608, found: 461.0600.

**1-(4-bromophenyl)-2-(2-(thiazol-2-yl)-4H-benzo[4,5] imidazo[1,2-b][1,2,4]triazol-4-yl)ethan-1-one 22c**

According to the general procedure C, the reaction was finished in 12 h.

White powder,1.3 mg, yield 77% ^**1**^**H NMR** (400 MHz, CDCl_3_) 7.97 (t, *J* = 1.2 Hz, 1H), 7.94–7.92 (m, 3H), 7.71 (d, *J* = 6.8 Hz, 2H), 7.44 (t, *J* = 1.6 Hz, 1H), 7.40–7.38 (m, 2H), 7.26–7.24 (m, 1H), 5.71 (s, 3H).

**1-(4-bromophenyl)-2-(2-(3-methylthiophen-2-yl)-4H-benzo[4,5] imidazo[1,2-b][1,2,4]triazol-4-yl)ethan-1-one 22d**

According to the general procedure C, the reaction was finished in 12 h.

White powder,3.7 mg, yield 83% ^**1**^**H NMR** (400 MHz, CDCl_3_) δ 7.95 (d, *J* = 8.4 Hz, 2H), 7.90 – 7.85 (m, 1H), 7.70 (d, *J* = 8.4 Hz, 2H), 7.33 (t, *J* = 3.6 Hz, 3H), 7.23 – 7.18 (m, 1H), 6.95 (d, *J* = 5.0 Hz, 1H), 5.66 (s, 2H), 2.69 (s, 3H).

^**13**^**C NMR** (151 MHz, CDCl_3_) δ 190.2, 142.1, 138.0, 134.9, 132.9, 132.5, 131.6, 129.9, 129.7, 128.3, 125.3, 124.8, 123.98, 122.28, 121.0, 111.38, 110.6, 49.7, 29.7, 15.9.

**HRMS** (ESI) [M + H]^+^ calculated for C_21_H_16_BrN_4_OS: 451.0223, found: 451.0224.

**1-(4-bromophenyl)-2-(2-(pyridin-2-yl)-4H-benzo[4,5] imidazo[1,2-b][1,2,4]triazol-4-yl)ethan-1-one 22e**

According to the general procedure C, the reaction was finished in 12 h.

White powder,2.7 mg, yield 54% ^**1**^**H NMR** (400 MHz, CDCl_3_) δ 8.76 (d, *J* = 4.4 Hz, 1H), 8.24 (d, *J* = 7.9 Hz, 1H), 7.97–7.90 (m, 3H), 7.81 (t, *J* = 7.7 Hz, 1H), 7.70 (d, *J* = 8.4 Hz, 2H), 7.40–7.31 (m, 3H), 7.23 (dd, *J* = 6.0, 3.1 Hz, 1H), 5.72 (s, 2H);

^**13**^**C NMR** (151 MHz, CDCl3) δ 190.0, 150.2, 150.0, 147.4, 136.7, 135.4, 132.8, 132.5, 129.9, 129.6, 124.8, 124.4, 124.0, 122.4, 121.8, 111.6, 110.7, 49.7.

**HRMS** (ESI) [M + H]^+^ calculated for C_21_H_15_BrN_5_O: 432.0454, found: 432.0468.

**2-(2-benzyl-4H-benzo[4,5]imidazo[1,2-b][1,2,4] triazol-4-yl)-1-(4-bromophenyl)ethan-1-one 22f**

According to the general procedure C, the reaction was finished in 12 h.

White powder,1.7 mg, yield 64% ^**1**^**H NMR** (400 MHz, CDCl3) δ 7.90 (d, *J* = 8.6 Hz, 2H), 7.81 (d, *J* = 9.1 Hz, 1H), 7.65 (d, *J* = 8.6 Hz, 2H), 7.39 (d, *J* = 7.0 Hz, 2H), 7.34–7.28 (m, 5H), 7.19–7.17 (m, 1H), 5.58 (s, 2H), 4.20 (s, 2H).

**1-(4-bromophenyl)-2-(2-(tert-butyl)-4H-benzo[4,5] imidazo[1,2-b][1,2,4]triazol-4-yl)ethan-1-one 22g**

According to the general procedure C, the reaction was finished in 12 h.

White powder,8.2 mg, yield 57% ^**1**^**H NMR** (400 MHz, CDCl_3_) δ 7.92 (d, *J* = 7.0 Hz, 2H), 7.79 (dd, *J* = 4.9, 2.3 Hz, 1H), 7.65 (d, *J* = 6.9 Hz, 2H), 7.27 (s, 1H), 7.25 (s, 1H), 7.19–7.10 (m, 1H), 5.60 (s, 2H), 1.43 (s, 9H);

^**13**^**C NMR** (151 MHz, CDCl_3_) δ 190.4, 176.1, 134.7, 132.9, 132.4, 129.8, 129.7,124.8, 123.5, 121.9, 111.0, 110.4, 49.7, 34.1, 29.7.

**HRMS** (ESI) [M + H]^+^ calculated for C_20_H_20_BrN_4_O: 411.0815, found: 411.0804.

**1-(4-bromophenyl)-2-(2-(5-(p-tolyl)thiophen-2-yl)-4H-benzo[4,5]imidazo[1,2-b][1,2,4]triazol-4-yl)ethan-1-one 25**

To a mixture of 16b (14.4 mg, 0.034 mmol), 23 (9.3 mg, 0.069 mmol), Pd(OAc)_2_ (0.4 mg, 0.0017 mmol), PPh_3_ (1.8 mg, 0.0069 mmol) were added ethanol and toluene. The mixture was then treated with sat. aq. NaHCO_3_. The reaction was heated at 80°C for 8 h then 100°C for 5 h. Then the reaction was cooled and concentrated in vacuo to yield the crude product and used directly in next step.

According to the general procedure C, the reaction was finished in 12 h.

White powder,8.0 mg, yield 65% ^**1**^**H NMR** (400 MHz, CDCl_3_) δ 7.93 (d, *J* = 8.4 Hz, 2H), 7.89–7.85 (m, 1H), 7.70 (dd, *J* = 6.1, 4.8 Hz, 3H), 7.56 (d, *J* = 8.1 Hz, 2H), 7.36–7.31 (m, 2H), 7.29 (d, *J* = 3.8 Hz, 1H), 7.23–7.17 (m, 3H), 5.66 (s, 2H), 2.38 (s, 3H);

^**13**^**C NMR** (151 MHz, CDCl_3_) δ 190.0, 145.8, 137.8, 135.0, 132.9, 132.8, 132.5, 131.3, 129.9, 129.7, 129.6, 127.5, 125.7, 124.7, 124.0, 123.2, 122.3, 111.3, 110.6, 49.7, 21.2.

**HRMS** (ESI) [M + H]^+^ calculated for C_27_H_20_BrN_4_OS: 527.0536, found: 527.0542.

**5-(4-(2-(4-bromophenyl)-2-oxoethyl)-4H-benzo[4,5]imidazo[1,2-b][1,2,4]triazol-2-yl)thiophene-2-carbonitrile 27**

To a solution of 16b (7.2 mg, 0.0172 mmol) in DMF was added CuCN (4.6 mg, 0.0515 mmol), the reaction was stirred at 160°C for 6 h, then cooled to r.t. Diluted with water and the resulting solution was stirred with saturated aq. NH_4_Cl/ NH_3_•H_2_O (9:1) solution for 15 min. Then extracted with ethyl acetate. The organic layer was dried over Na_2_SO_4_, filtered and concentrated in vacuo to yield the crude product and used directly in next step.

According to the general procedure C, the reaction was finished in 12 h.

White powder,4.9 mg, yield 61% ^**1**^**H NMR** (400 MHz, CDCl_3_) δ 7.94 (d, *J* = 8.6 Hz, 2H), 7.74 – 7.70 (m, 3H), 7.61 (d, *J* = 4.0 Hz, 1H), 7.49 (d, *J* = 8.6 Hz, 1H), 7.38 (dd, *J* = 6.0, 3.3 Hz, 2H), 6.99 (d, *J* = 4.6 Hz, 1H), 5.67 (s, 2H);

**HRMS** (ESI) [M + H]^+^ calculated for C_21_H_13_BrN_5_OS: 462.0019, found: 462.0020.

**1-(4-bromophenyl)-2-(2-(5-chlorothiophen-2-yl)-4H-benzo[4,5] imidazo[1,2-b][1,2,4]triazol-4-yl)ethan-1-one 29**

To a solution of 16b (6.5 mg, 0.016 mmol) in DMF was added CuCl (7.7 mg, 0.078 mmol), the reaction was stirred at 160°C for 6 h, then cooled to r.t. Ethyl acetate was added and the resulting solution was stirred with saturated aq. NH_4_Cl/ NH_3_•H_2_O (9:1) solution for 15 min. Then extracted with ethyl acetate. The organic layer was dried over Na_2_SO_4_, filtered and concentrated in vacuo to yield the crude product and used directly in next step.

According to the general procedure C, the reaction was finished in 12 h.

White powder,7.6 mg, yield 32% ^**1**^**H NMR** (400 MHz, CDCl_3_) δ 7.94 (d, *J* = 8.4 Hz, 2H), 7.89–7.83 (m, 1H), 7.71 (d, *J* = 8.4 Hz, 2H), 7.52 (d, *J* = 3.9 Hz, 1H), 7.40–7.32 (m, 2H), 7.23–7.17 (m, 1H), 6.93 (d, *J* = 3.9 Hz, 1H), 5.65 (s, 2H);

^**13**^**C NMR** (151 MHz, CDCl_3_) δ 189.9, 135.0, 133.1, 132.7, 132.5, 131.4, 129.9, 129.7, 126.9, 125.7, 125.2, 124.6, 124.2, 122.5, 111.3, 110.6, 49.60.

**HRMS** (ESI) [M + H]^+^ calculated for C_20_H_13_BrClN_4_OS: 470.9676, found: 470.9662

**1-(4-hydroxyphenyl)-2-(2-(thiophen-2-yl)-4H-benzo[4,5]imidazo[1,2-b][1,2,4]triazol-4-yl)ethan-1-one 7h**

To a solution of 7b (4.3 mg, 0.011 mmol) in 0.8 mL of CH_2_Cl_2_ at 0°C was added BBr_3_ (1.0 M in CH_2_Cl_2_, 55 μL, 0.055 mmol) dropwise. The resulting solution was stirred for 14 h from 0 to 15°C gradually and quenched with saturated aqueous Na_2_CO_3_ (5 mL). The mixture was extracted with CHCl_3_/*i-*PrOH (3/1) three times, and the combined organic layers were dried over anhydrous Na_2_SO_4_, filtered and concentrated in vacuo. Flash chromatography (THF/DCM = 3/97) provided 7 h (4.1 mg, 34%) as a white solid.

^**1**^**H NMR** (400 MHz, CDCl_3_) δ 7.91 (d, *J* = 4.7 Hz, 1H), 7.80 (d, *J* = 3.4 Hz, 1H), 7.63 (d, *J* = 8.8 Hz, 2H), 7.42 – 7.35 (m, 4H), 7.14 – 7.12 (m, 1H), 6.90 (d, *J* = 8.5 Hz, 2H), 5.54 (s, 2H);

**HRMS** (ESI) [M + H]^+^ calculated for C_20_H_15_N_4_O_2_S: 375.0910, found: 375.0913.

**1-(4-aminophenyl)-2-(2-(thiophen-2-yl)-4H-benzo[4,5]imidazo[1,2-b][1,2,4]triazol-4-yl)ethan-1-one 7i**

Methanol solution of 7c (7.9 mg, 0.0196 mmol) and 5% Pd/C (1.6 mg) was stirred vigorously for 10 h under H_2_ atmosphere. The insoluble material was removed by filtration through celite, the filtrate was concentrated in vacuo. Flash chromatography (MeOH/DCM = 3/97) provided 7i (1.2 mg, 16%) as a light yellow solid.

^**1**^**H NMR** (400 MHz, CDCl_3_) δ 7.89 (d, *J* = 8.2 Hz, 3H), 7.77 (s, 1H), 7.37 (d, *J* = 4.7 Hz, 1H), 7.31 (s, 2H), 7.23 (s, 1H), 7.12 (s, 1H), 6.70 (d, *J* = 8.2 Hz, 2H), 5.61 (s, 2H), 4.31 (s, 2H).

**4-(2-(2-(thiophen-2-yl)-4H-benzo[4,5]imidazo[1,2-b][1,2,4]triazol-4-yl)acetyl)benzoic acid 7j**

To a solution of 7d (4.3 mg, 0.0103 mmol) in a mixture of methanol (0.1 mL), water (0.1 mL) and THF (0.3 ml) was added LiOH (4.3 mg, 0.103 mmol) and refluxed for 1 h. After the reaction mixture cold to room temperature, 1M HCl was added, and extracted with EtOAc. The extracted organic layer was dried over anhydrous Na_2_SO_4_, concentrated in vacuo. Flash chromatography (MeOH/DCM = 3/97) provided 7j (4.2 mg, 36%) as a white solid.

^**1**^**H NMR** (400 MHz, CD_3_OD) δ 8.23 (d, *J* = 2.8 Hz, 4H), 7.88 (t, *J* = 4.8 Hz, 1H), 7.75–7.74 (m, 1H), 7.53 (dd, *J* = 5.2, 1.2 Hz, 2H), 7.42 (t, *J* = 4.8, 2H), 7.16–7.14 (m, 1H), 6.03 (s, 2H).

^**13**^**C NMR** (151 MHz, CDCl_3_) δ 190.3, 163.7, 158.4, 145.3, 142.6, 138.1, 133.0, 127.2, 125.9, 125.3, 124.5, 123.9, 123.8, 123.2, 122.7, 121.5, 111.6, 110.0, 51.9.

**HRMS** (ESI) [M + H]^+^ calculated for C_21_H_15_N_4_O_3_S: 403.0859, found: 403.0857;

General procedure D: To a solution of 22c (1.0 eq) in DMF (0.33 M) was added DIPEA (7.0 eq) and stirred for 1 h. In a separate flask 30 (2.0 eq), EDCI (3.0 eq), HOBt (3.0 eq), and DMF (1.0 M) are mixed and stirred for 1 h. The contents of the second flask are transferred via canula into the first flask and the reaction is allowed to stirred for 24 h. The reaction was diluted with EtOAc /THF (10/1) and washed with saturated aqueous NH_4_Cl and brine. The organic layer was dried over Na_2_SO_4_, filtered and concentrated in vacuo to yield the crude product. The crude product was purified by flash chromatography (PE/EtOAc = 2/1) to yield the product.

**(4-bromophenyl) (2-(thiazol-2-yl)-4H-benzo[4,5] imidazo[1,2-b][1,2,4]triazol-4-yl)methanone 31a**

According to the general procedure D, the reaction was finished in 24 h.

White powder,3.9 mg, yield 25% ^**1**^**H NMR** (400 MHz, CDCl_3_) δ 8.43–8.36 (m, 1H), 7.88–7.83 (m, 3H), 7.72 (d, *J* = 8.6 Hz, 2H), 7.63 (dd, *J* = 3.6, 1.1 Hz, 1H), 7.53–7.44 (m, 2H), 7.36 (dd, *J* = 5.0, 1.1 Hz, 1H), 7.08 (dd, *J* = 5.0, 3.7 Hz, 1H).

**HRMS** (ESI) [M + H]^+^ calculated for C_19_H_12_BrN_4_OS: 422.9910, found: 422.9911.

**2-(4-bromophenyl)-1-(2-(thiazol-2-yl)-4H-benzo[4,5] imidazo[1,2-b][1,2,4]triazol-4-yl)ethan-1-one 31b**

According to the general procedure D, the reaction was finished in 24 h.

White powder,2.1 mg, yield 52% ^**1**^**H NMR** (400 MHz, CDCl_3_) δ 8.50 (d, *J* = 7.7 Hz, 1H), 7.85 (dd, *J* = 3.6, 1.1 Hz, 1H), 7.83–7.78 (m, 1H), 7.50 (d, *J* = 8.4 Hz, 2H), 7.48–7.42 (m, 3H), 7.40 (d, *J* = 8.4 Hz, 2H), 7.17 (dd, *J* = 5.0, 3.7 Hz, 1H), 4.75 (s, 2H).

**HRMS** (ESI) [M + H]^+^ calculated for C_20_H_14_BrN_4_OS: 437.0066, found: 437.0065.

**3-(4-bromophenyl)-1-(2-(thiazol-2-yl)-4H-benzo[4,5] imidazo[1,2-b][1,2,4]triazol-4-yl)propan-1-one 31c**

According to the general procedure D, the reaction was finished in 24 h.

White powder,12.8 mg yield 57% ^**1**^**H NMR** (400 MHz, DMSO-*d*_6_) δ 8.44 (dd, *J* = 6.4, 2.7 Hz, 1H), 7.92 (dd, *J* = 6.2, 2.8 Hz, 1H), 7.79–7.69 (m, 2H), 7.62–7.46 (m, 4H), 7.38 (d, *J* = 8.4 Hz, 2H), 7.22 (dd, *J* = 4.9, 3.7 Hz, 1H), 3.66 (t, *J* = 7.6 Hz, 2H), 3.09 (t, *J* = 7.6 Hz, 2H).

^**13**^**C NMR** (151 MHz, CDCl3) δ 169.9, 161.6, 139.0, 133.7, 132.1, 131.6, 130.4, 130.3, 127.8, 127.4, 127.2, 125.8, 125.6, 125.4, 120.3, 118.1, 110.7, 38.4, 29.6.

**HRMS** (ESI) [M + H]^+^ calculated for C_21_H_16_BrN_4_OS: 451.0223, found: 451.0219.

General procedure for bromination: To a mixture of 4 (1.0 eq) in CHCl_3_ (0.1 M), was added N-bromosuccinimide (NBS) (1.03 eq). The reaction was stirred at r.t. for 4 h. Then the reaction was quenched with saturated aq. NaHCO_3_ and extracted with ethyl acetate, the organic layer was dried over Na_2_SO_4_, filtered and concentrated in vacuo to yield the crude product. The residue was redissolved in CH_2_Cl_2_. Et_3_N (2.5 eq) and (Boc)_2_O (3.7 eq) was added sequentially. The resulting reaction mixture was stirred at r.t. overnight then concentrated and purified by flash chromatography (PE/ CH_2_Cl_2_ = 7/3) to yield the product.

**tert-butyl7-bromo-2-(thiophen-2-yl)-4H-benzo[4,5] imidazo[1,2-b] [1,2,4]triazole-4-carboxylate 16a**

According to the general procedure for bromination, the reaction was finished in 12 h.

White powder,9.3 mg, yield 7%^**1**^**H NMR** (400 MHz, CDCl3) δ 8.02 (d, *J* = 8.8 Hz, 1H), 7.95 (d, *J* = 1.9 Hz, 1H), 7.58 (d, *J* = 3.9 Hz, 1H), 7.53 (dd, *J* = 8.8, 1.9 Hz, 1H), 7.07 (d, *J* = 3.9 Hz, 1H), 1.75 (s, 9H).

**tert-butyl2-(5-bromothiophen-2-yl)-4H-benzo[4,5] imidazo[1,2-b] [1,2,4]triazole-4-carboxylate 16b**

According to the general procedure for bromination, the reaction was finished in 12 h.

White powder,23.1 mg, yield 17% ^**1**^**H NMR** (400 MHz, CDCl3) δ 8.14 (dd, *J* = 5.8, 3.2 Hz, 1H), 7.82–7.77 (m, 1H), 7.59 (d, *J* = 3.9 Hz, 1H), 7.46 – 7.39 (m, 2H), 7.07 (d, *J* = 3.9 Hz, 1H), 1.76 (s, 10H).

**tert-butyl7-bromo-2-(5-bromothiophen-2-yl)-4H-benzo[4,5] imidazo[1,2-b][1,2,4]triazole-4-carboxylate 16c**

According to the general procedure for bromination, the reaction was finished in 12 h.

White powder,70.7 mg, yield 44% ^**1**^**H NMR** (400 MHz, CDCl_3_) δ 8.02 (d, *J* = 8.8 Hz, 1H), 7.95 (d, *J* = 1.9 Hz, 1H), 7.58 (d, *J* = 3.9 Hz, 1H), 7.53 (dd, *J* = 8.8, 1.9 Hz, 1H), 7.07 (d, *J* = 3.9 Hz, 1H), 1.75 (s, 9H).

### Compound Effective Assay

Procedure for cell culture and establishment of the reporter cell line were reported in our previously published paper (Li et al., 2018).

Before plating cells, 384-well plates (ViewPlate, PerkinElmer) were coated with PDL (poly-D-lysine, Sigma). For each well, 1,300 B2-17 cells were plated by using a Matrix Wellmate microplate dispenser (Thermo Scientific). Then, 0.25 μL of compound was added to each well by using a versatile pipetting robot, the Biomek FX workstation (Beckman Coulter), to give a final compound concentration of 5 μM. After treatment for 72 h, the cell nuclei were stained by Hoechst 33342 (Sigma) before imaging.

The cells were imaged with Opera LX (PerkinElmer, three views per well by using 10 x (air) objective lens). Images were analyzed with the Columbus system, the mean percentage of EGFP+ cells (RGFP) was obtained to extrapolate the number of EGFP expressing cells and Hoechst-stained nuclei.

For the titration assay, the positive compounds were diluted in DMSO at concentrations ranging from 0.625 to 4 mM. Then, the B2-17 cells were treated by gradient compound and imaged as described before.

For each compound, a dose-response series was generated by using Origin 6.0 Pro software. The dot plot was fit by a DoseResp curve and then the EC50 was calculated.

### Viability Assays

HEK 293 F cells were plated in 384-well, flat-bottom, tissue culture dishes (Corning) and treated with compounds, incubated 3 days, and assayed for viability using the CellTiter-Glo Luminescent Cell Viability Assay (Promega) according to the manufacturer's protocol. The absorbance at 490 and 650 nm (reference) was measured with an EnSight plate reader (Perkin Elmer). Data were normalized to the untreated control (100% viability). Each treatment was tested in two independent assays, each containing 3 replicates. The titration-viability curves of each compound were also generated by using Origin 6.0 Pro software.

## Results and Discussion

**Scheme 2:**
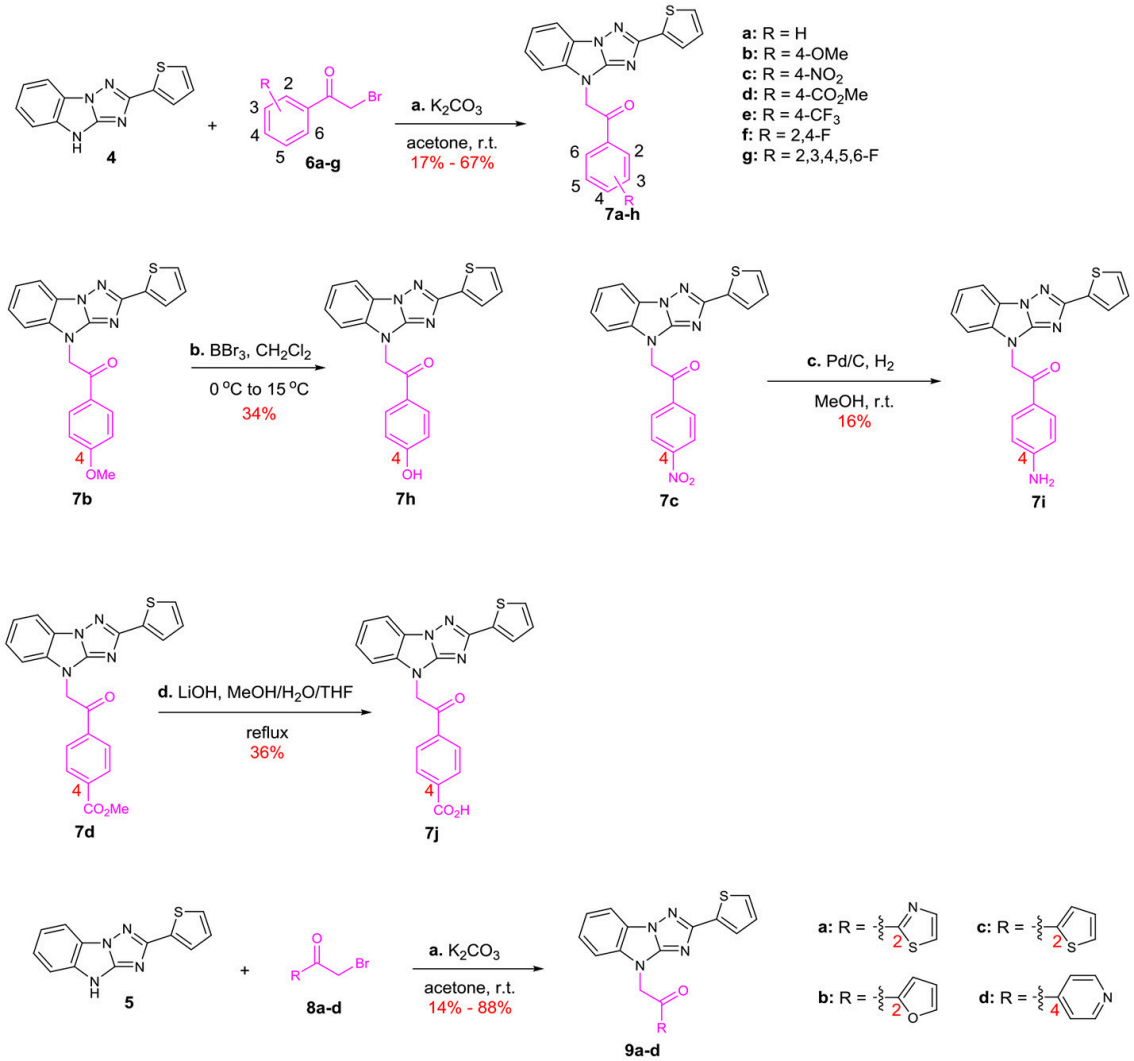
Syntheses of the C-modified analogs: (a) K_2_CO_3_, acetone, r.t. (b) BBr_3_, CH_2_Cl_2_, 0–15°C (c) Pd/C, H_2_, MeOH, r.t. (d) LiOH, MeOH/H_2_O/THF, reflux.

We started with the structural diversification of C. The syntheses of C analogs are described in Scheme 2. N-alkylation of key intermediate **4** with bromide **6** bearing different substituents on the benzene ring provided **7a**-**g**, including unsubstituted, 4-OMe, 4-NO_2_, 4-CO_2_Me, 4-CF_3_, 2,4-F and 2,3,4,5,6-F groups. Similarly, N-alkylation with 2-bromo-1-heterocyclic ethanone **8a-d** afforded skeletons **9a-d** with various heterocyclic substituents, including 2-thiazolyl, 2-furyl, 2-thiophenyl and 4-pyridyl groups. Meanwhile, methoxyl substituted compound **7b** was demethylated by using BBr_3_ to provide phenolic compound **7h**. The nitro group of **7c** was reduced to afford aniline **7i**, and 4-benzoic acid **7j** was derived from methyl ester **7d** by hydrolysis using LiOH.

**Scheme 3:**
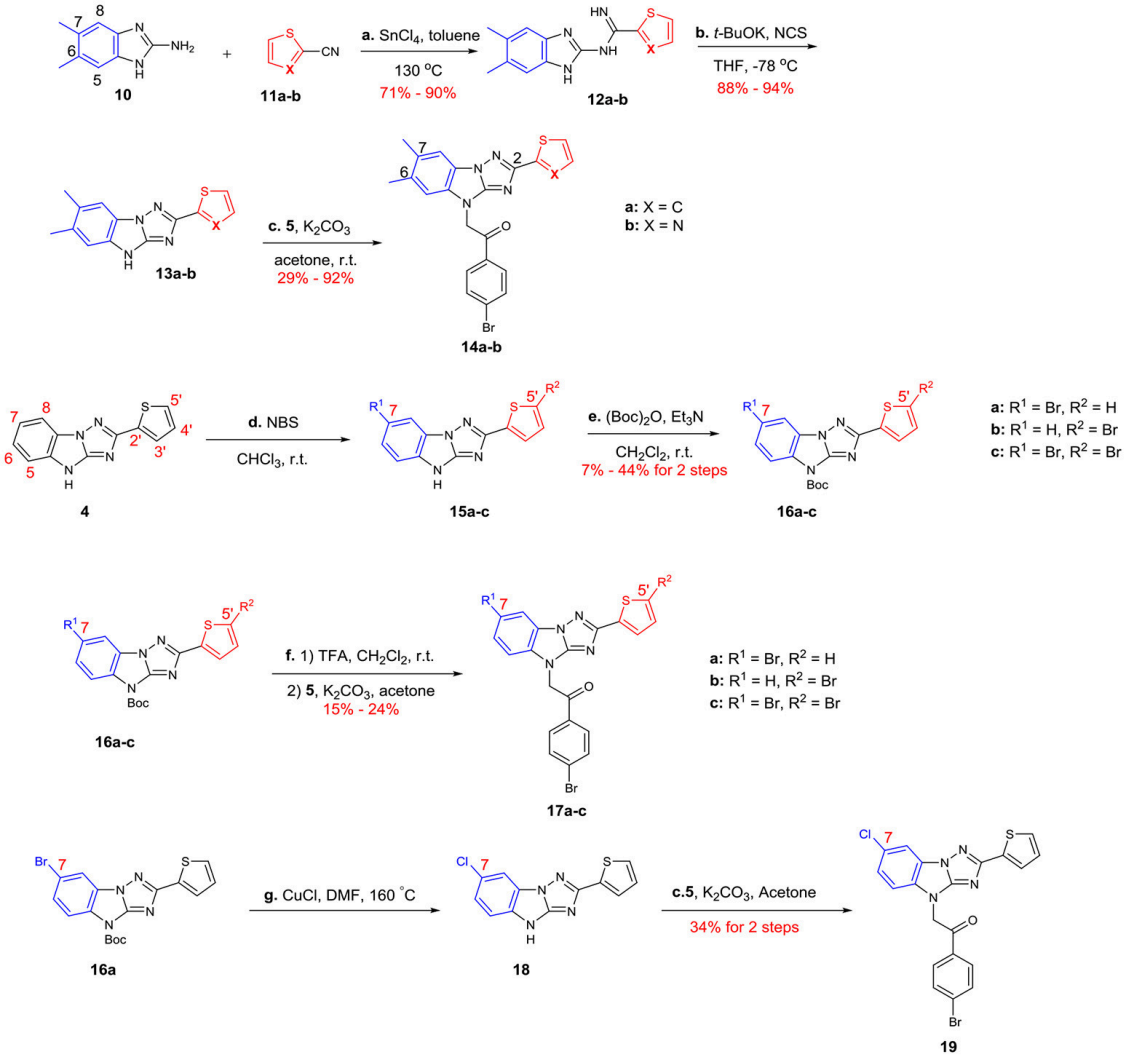
Syntheses of the A-modified analogs: (a) SnCl_4_, toluene, 130°C (b) *t*-BuOK, NCS, THF, −78°C (c) K_2_CO_3_, acetone, r.t. (d) NBS, CHCl_3_, r.t. (e) (Boc)_2_O, Et_3_N, CH_2_Cl_2_, r.t. (f) 1) TFA, CH_2_Cl_2_, r.t. 2) 6, K_2_CO_3_, acetone (g) CuCl, DMF, 160°C NBS = N-Bromosuccinimide (Boc)_2_O = Di-tert-butyl dicarbonate Et_3_N = Triethylamine TFA = Trifluoroacetic acid.

The syntheses of A analogs are summarized in Scheme 3. Both 6,7-dimethyl-2-thiophenyl (**14a**) and 6,7-dimethyl-2-thiazolyl (**14b**) analogs were synthesized using the method we describe in Scheme 1, where building blocks were changed correspondingly (**10** and **11a**-**b**). To prepare 7-halogenated analogs, the key intermediate **4** was brominated using NBS, but the regioselectivity for this reaction was not good, which provided **15a**-**c** as a mixture. Due to the similar polarity of these three compounds, it was difficult to separate them. Fortunately, after the imidazole of **15a**-**c** was protected with Boc, the generated compounds **16a**-**c** were easily separated. **16a**-**c** was then deprotected and alkylated with 2,4′-dibromoacetophenone **5** to give analogs **17a**-**c**, respectively. In addition, **16a** was chlorinated by using CuCl, and the subsequent substitution reaction provided **19**.

**Scheme 4:**
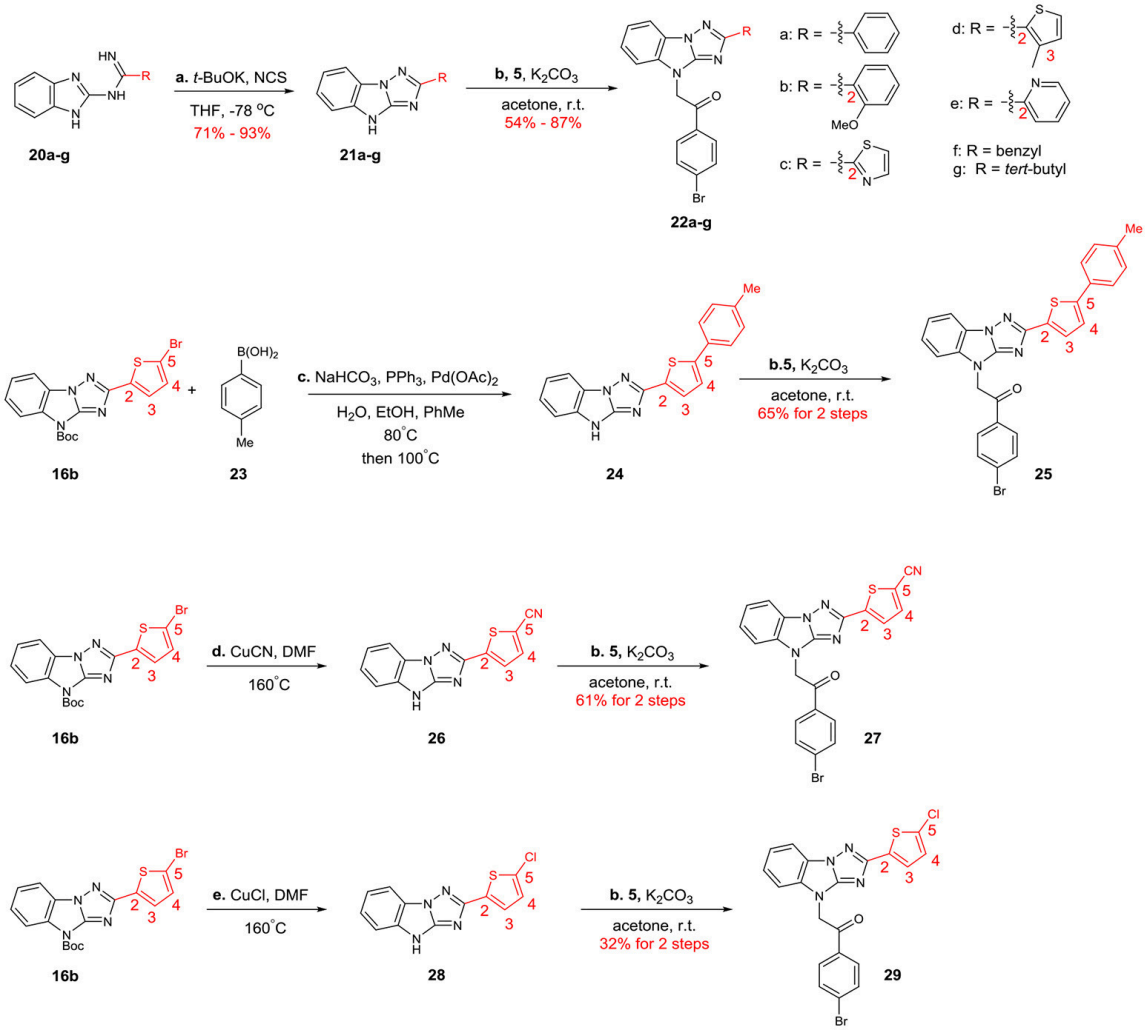
Syntheses of the B-modified analogs: (a) *t*-BuOK, NCS, THF, −78°C (b) K_2_CO_3_, acetone, r.t. (c) NaHCO_3_, PPh_3_, Pd(OAc)_2_, H_2_O, EtOH, PhMe, 80°C; then 100°C (d) CuCN, DMF, 160°C (e) CuCl, DMF, 160°C.

The structural diversification of B is summarized in Scheme 4. **22a**-**g**, whose B were phenyl, 2-methoxyphenyl, 2-thiazolyl, 3-methylthiophen-2-yl, 2-pyridyl, benzyl and *tert*-butyl groups, were prepared through the same 2-step procedures. Then **16b** was utilized as a key intermediate for the synthesis of **25**, **27** and **29**. Suzuki coupling of **16b** and subsequent N-alkylation reaction provided analog **25**. **27** and **39** were obtained through cyanation and chlorination reactions followed by N-alkylation reaction.

**Scheme 5:**
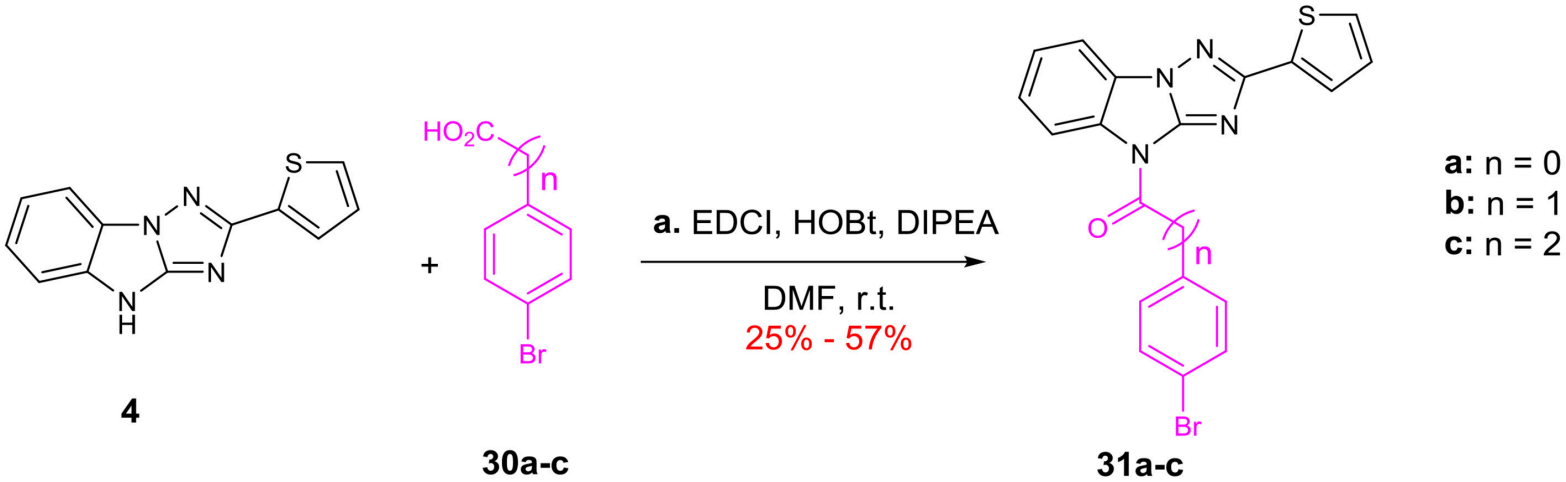
Change of the length for the C linker: (a) EDCI, HOBt, DIPEA, DMF, r.t. EDCI = 1-(3-Dimethylaminopropyl)-3-ethylcarbodiimide HOBt = 1-Hydroxybenzotriazole DIPEA = N,N-Diisopropylethylamine.

Finally, modifications of the length of the linker are summarized in Scheme 5. **4** were condensed with acids **30a**-**c** by using EDCI and HOBt afford amides **31a**-**c**, which bearing one- to three-carbon linker.

After constructing the DOS library containing over 30 compounds based on the central imidazo[1,2-*b*][1,2,4]triazole of **LX-3**, we set out to investigate the bioactivities of those compounds using the same screening method as we used on LX-1 to LX-7 to compare with the lead compound LX-3(**Compound effective assay and Viability assays**). The C analogs were first evaluated (Table 2). Interestingly, we found that bromine atoms were important for activity, since when bromine atoms were removed (compound **7a**), the activity of derepressing the EGFP reporter gene silenced by DNA methylation was lost. When the bromine atoms were replaced with other hydrophobic substituent such as methoxy group (compound **7b**), nitro group (compound **7c**) or hydrophilic groups such as hydroxyl group (compound **7h**), amino group (compound **7i**) and carboxyl group (compound **7j**), the activity was decreased to some extent. However, hydrophobic groups were more beneficial, because when the carboxyl group (compound **7j**) was protected as methyl ester (compound **7d**), the activity was significantly increased (EC_50_ = 2.4 μM). Compound **7d** showed even better activity and less toxicity (IC_50_ = 10.2 μM) than the hit compound **LX-3**. When we continued to replace bromine atoms with other halogen or halogen containing groups, such as trifluoromethyl groups (compound **7e**), the activity remained the same. However, when halogen atoms (compounds **7f** and **7g**) were introduced into the 2-, 4- position (*ortho)* or 3-, 5- position (*meta)*, the potency was significantly decreased. Next, the effect of replacement of C benzene ring with different heterocyclic rings (compound **9a-d**) including thiazole, furan, thiophene and pyridine was also evaluated. It was found that the activity was comparable when the thiophene ring was introduced, even though the maximal R_GFP_ (40%) was lower than **LX-3**, while other heterocycles caused reduced activity to varying degrees.

**Table 2:** SAR of the substitutions on C.

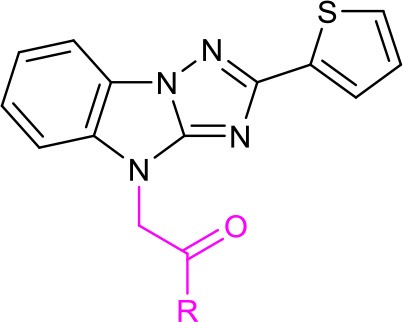				
**No**.	**R**	**EC**_**50**_ **(μM)**	**Maximal R**_**GFP**_%	**IC**_**50**_ **(μM)**
LX-3		2.8	79	5.1
7a	phenyl	–	7	1.9
7b	4-methoxyphenyl	6.2	23	3.1
7c	4-nitrophenyl	14.9	41	11.9
7d	4-(methoxycarbonyl)phenyl	2.4	73	10.2
7e	4-trifluoromethyphenyl	3.7	81	4.7
7f	2,4-difluorophenyl	–	16	17.8
7g	2,3,4,5,6-pentafluorophenyl	–	10	1.1
7h	4-hydroxyphenyl	5.8	56	16.7
7i	4-aminophenyl	–	19	5.3
7j	4-carboxyphenyl	–	7	4.8
9a	2-thiazolyl	4.7	30	8.7
9b	2-furyl	–	6.6	–
9c	2-thiophenyl	2.3	40	4.9
9d	4-pyridyl	13.6	35	6.5

Then, we focused on the structure-activity relationship of A (Table 3). When hydrophobic groups, such as halogens and methyl group were installed at the 6- or 7-position (compounds **14a**, **17a**, **19**), the activities were significantly decreased. Moreover, we found that the water solubility of these derivatives decreased a lot. However, when B was replaced with thiazole (**14b** vs. **14a**), the solubility significantly increased and the activity was comparable with the hit compound **LX-3**.

**Table 3:** SAR of the substitutions on A.

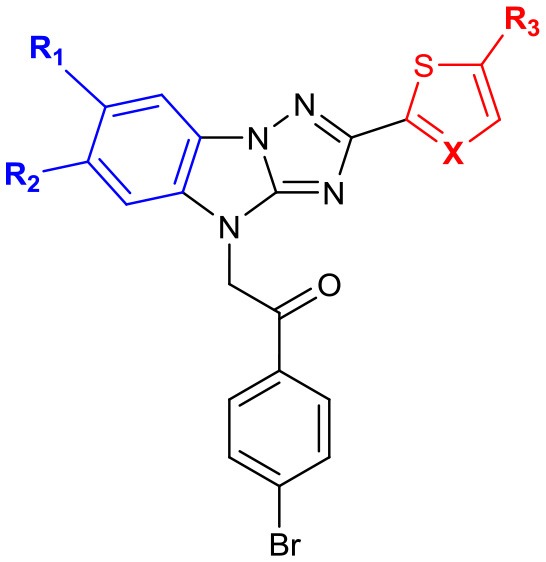							
**No**.	**R**_**1**_	**R**_**2**_	**R**_**3**_	**X**	**EC**_**50**_ **(μM)**	**Maximal R**_**GFP**_%	**IC**_**50**_ **(μM)**
LX-3					2.8	79	5.1
14a	CH_3_	CH_3_	H	C	28.0	26	3.9
14b	CH_3_	CH_3_	H	N	3.4	58	2.1
17a	Br	H	H	C	19.4	41	6.6
17c	Br	H	Br	C	21.8	28	3.8
19	Cl	H	H	C	14.9	63	14.3

Next, we evaluated the effect of B on the activity of derepressing the EGFP reporter gene silenced by DNA methylation (Table 4). When the thiophene ring was replaced by benzene ring or substituted benzene ring (compound **22a**,**b**) the activity decreased significantly or even disappeared, indicating that it was not feasible to replace B with aryl groups. Other heterocyclic rings, including thiazole and pyridine were also evaluated. Pyridine led to the loss of activity, while thiazole could slightly reduce activity (EC_50_ = 4.5 μM). Further replacing thiophene with benzyl (compound **22f**) or *tert*-butyl group (compound **22g**) also significantly decreased or even lost the activity (maximal R_GFP_ was <22%). Therefore, we further evaluated the effect of different substituents on thiophene ring. When methyl group was introduced to thiophene 3-position (**22d**), the activity disappeared. The introduction of aryl ring (compound **25**) and cyano group (compound **27**) at the 5-position of thiophene resulted in significant decrease or disappearance of activity, while the introduction of chlorine atom (compound **29**) resulted in little reduction of potency. This indicates that the substitution of 3- or 5-position on thiophene ring cannot improve the activity.

**Table 4:** SAR of the substitutions on B.

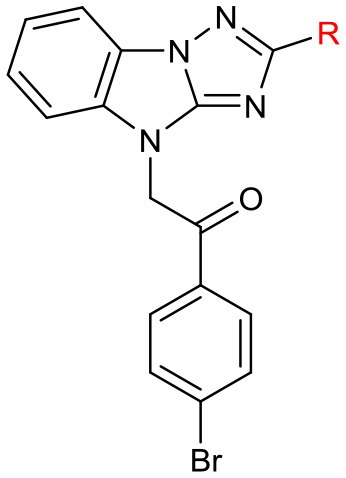				
**No**.	**R**	**EC**_**50**_ **(μM)**	**Maximal R**_**GFP**_%	**IC**_**50**_ **(μM)**
LX-3		2.8	79	5.1
22a	phenyl	–	6%	4.4
22b	2-methoxyphenyl	6.8	21	3.3
22c	2-thiazolyl	4.5	48	5.3
22d	3-methylthiophen-2-yl	–	19	12.3
22e	2-pyridyl	–	6	2.8
22f	benzyl	6.4	22	4.7
22g	*tert*-butyl	–	10	8.3
25	5-(4-tolyl)thiophen-2-yl	29.9	61	10.9
27	5-cyanothiophen-2-yl	–	6	–
29	5-chlorothiophen-2-yl	8.9	83	13.63

We also evaluate the effect of the linker of C (Table 5). First, we reduced the two-carbon unit to a single-carbon unit (compound **31a**) and found that its activity of derepressing the DNA methylation silenced genes disappeared. The loss of activity can also be caused by exchanging relative location of the carbonyl and methylene groups (compound **31b**) or by increasing the carbon chain (compound **31c**). These results suggest that the two carbon units in the linker section are necessary for the activity, and that the carbonyl group must be on the side close to C.

**Table 5:** SAR of the linkers on C.

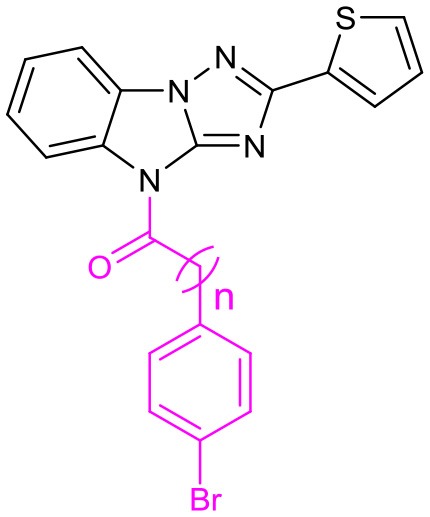				
**No**.	**n**	**EC**_**50**_ **(μM)**	**Maximal R**_**GFP**_ %	**IC**_**50**_ **(μM)**
LX-3		2.8	79	5.1
31a	0	–	4.0	7.8
31b	1	–	6.0	11.5
31c	2	–	9.0	15.9

**Figure 4:**
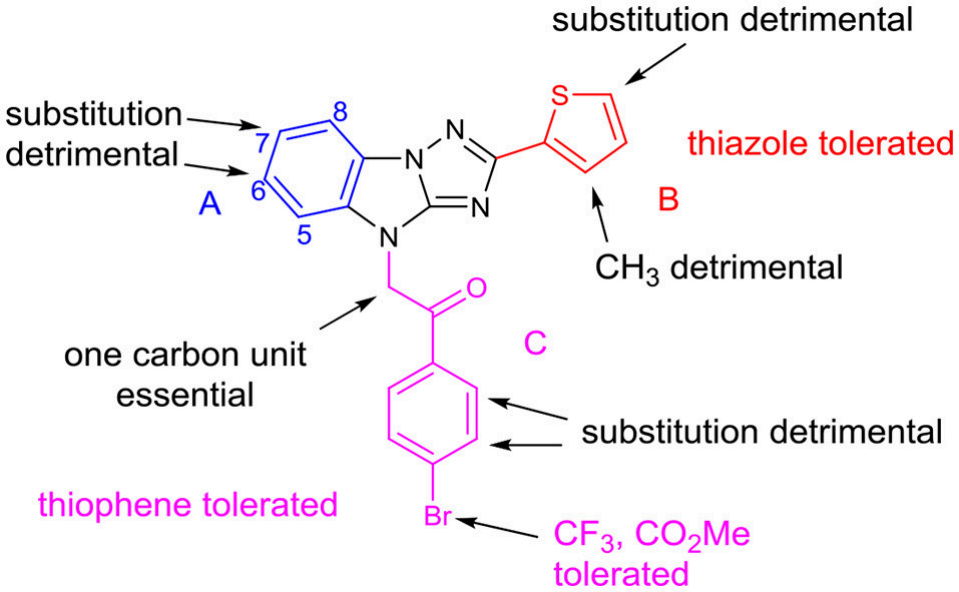
Summary of SAR.

Based on the above structural modification and biological evaluation of each part of the hit compound **LX-3**, we can conclude and draw the structure-activity relationship diagram (Figure 4). First, for A, 6- and 7-substitution will result in the reduced activity. And when B is thiazole, the 7-hydrophobic small group substitution is tolerable. Secondly, for B, replacing thiophene with thiazole slightly affects the activity, but when the 3- or 5-position of thiophene is substituted, the activity decreased. In C, when the bromine atoms were replaced by hydrophobic trifluoromethyl group or the methyl carboxylate, activity was retained well. Introducing substituents to the *ortho* or *meta* position of halogen significantly reduced the activity. When the benzene ring was replaced by thiophene ring, the activity was slightly reduced.

## Conclusion

Three small molecules, LX-3, LX-4, and LX-5, are identified utilizing high through put screening. Capable of activating a DNA methylation-repressed reporter gene, the activity can be achieved without any direct impact on DNA methylation machinery or factors downstream to DNA methylation. The derepression effect of compound LX-3, LX-4, and LX-5 functions through the activation of p38 MAPK pathway. Though capable of being activated by a number of extracellular stimuli (anisomycin, UV light, LPS, TNF-α, other cytokines and growth factors; Ono and Han, 2000; Chang and Karin, 2001; Zarubin and Jiahuai, 2005), p38 MAPK cannot be selectively activated. This lack of selectivity comes from the ability of p38 and diverse MAPKs module, especially c-jun N-terminal Kinases (JNK) module, to respond to similar stimuli. For example, anisomycin activates both p38 and JNK (Laird, 2003). Thus, an agonist that can selectively activate the p38 pathway is currently lacking. Deeper understanding of MAPKs is needed due to their involvement in many cellular processes, in which they are activated by diverse upstream stimuli(Meehan et al., 1992; Eden et al., 1998; De Smet et al., 1999). Compound(s) with specificity would serve this purpose well by facilitating the study toward individual pathways involving MAPKs. In our previous work (Li et al., 2018), we have already proved that Compound LX-3, LX-4 and LX-5 could selectively activate the p38 MAPK pathway in multiple cell types, these compounds can serve as a useful tool to discriminate the p38 module from other MAPK modules, thus they will hopefully play a role in the future applications.

However, with the exact targets of these compounds remaining to be known, futher optimization of our identified lead compounds is necessary. Through a series of modular syntheses based on the central imidazo[1,2-*b*][1,2,4]triazole structure of LX-3, a DOS library consisting of more than 30 derivatives was obtained. The SAR of A, B, C, and linker parts of the hit compound LX-3 was systematically evaluated, and the overall structure-activity diagrams were obtained. Through these endeavors, an analog, compound **7d** with slightly better activity of derepressing the EGFP reporter gene silenced by DNA methylation and less toxicity was identified. This work provides a general approach to efficiently access diverse heterocyclic molecules with interesting epigenetic modulation activities and set up a foundation for the subsequent design of chemical probes to identify the cellular targets of these lead compounds.

## Author Contributions

FW and JZ synthesized all of the compounds with the help of ES and JuZ. XL and BZ performed the biological evaluations of these compounds. XL supervised this work and wrote the paper with the help of FW and JZ.

### Conflict of Interest Statement

The authors declare that the research was conducted in the absence of any commercial or financial relationships that could be construed as a potential conflict of interest.
